# Supplementary material for: Telomere-to-telomere genome assembly of a male goat reveals variants associated with cashmere traits
Source: Nat Commun. 2024 Nov 20;15:10041. doi: 10.1038/s41467-024-54188-z (PMC11579321; doi:10.1038/s41467-024-54188-z)
Supplement: Supplementary file 1 — Supplementary Information [file 41467_2024_54188_MOESM1_ESM.pdf]

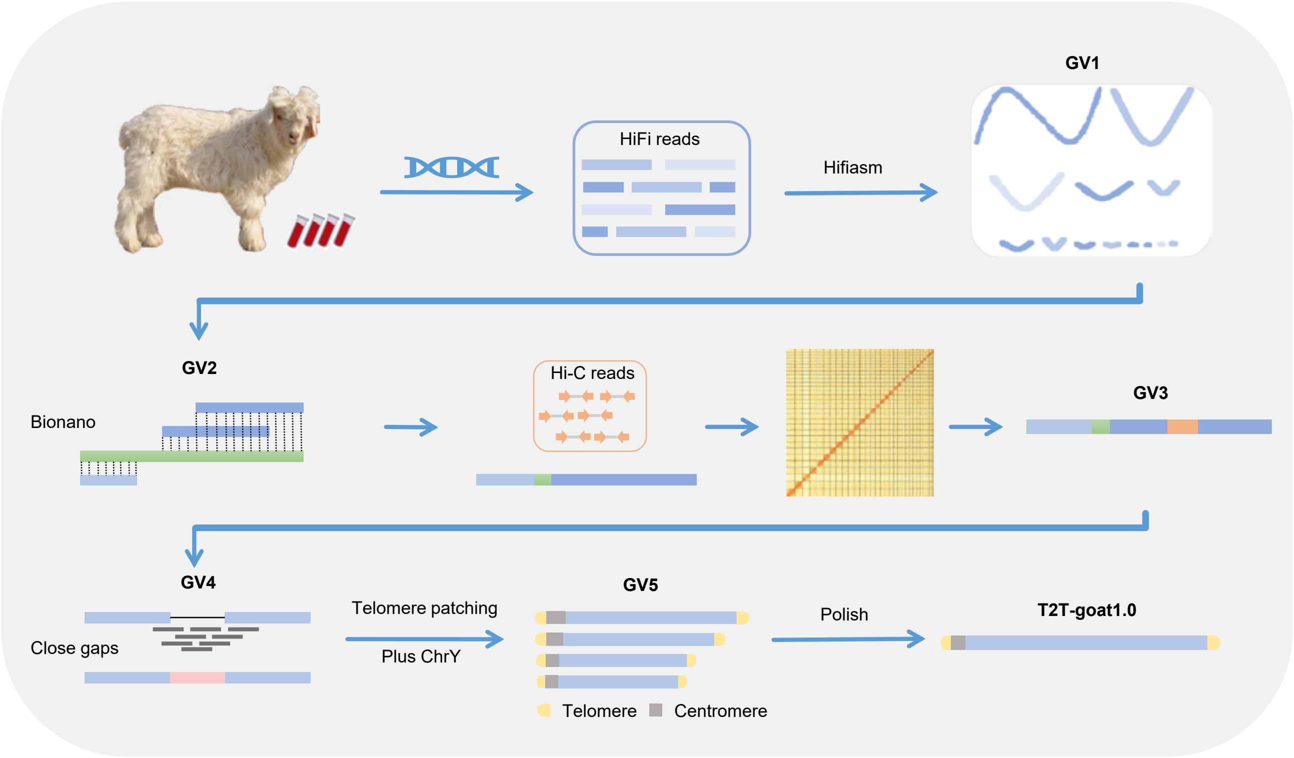

Supplementary Fig. 1 Genome assembly strategy for T2T-goat1.0. GV1~GV5 assemblies were created for T2T-goat1.0. GV1 is the initial assembly based on HiFi reads using Hifiasm software. Bionano scaffolding was performed to generate GV2, and Hi-C placed the contigs/scaffold onto the pseudochromosomes for GV3. Gaps were filled using ONT ultralong reads for GV4. Telomeres and chromosome Y independently assembled were added to GV5, which was polished for high-quality of T2T-goat1.0.

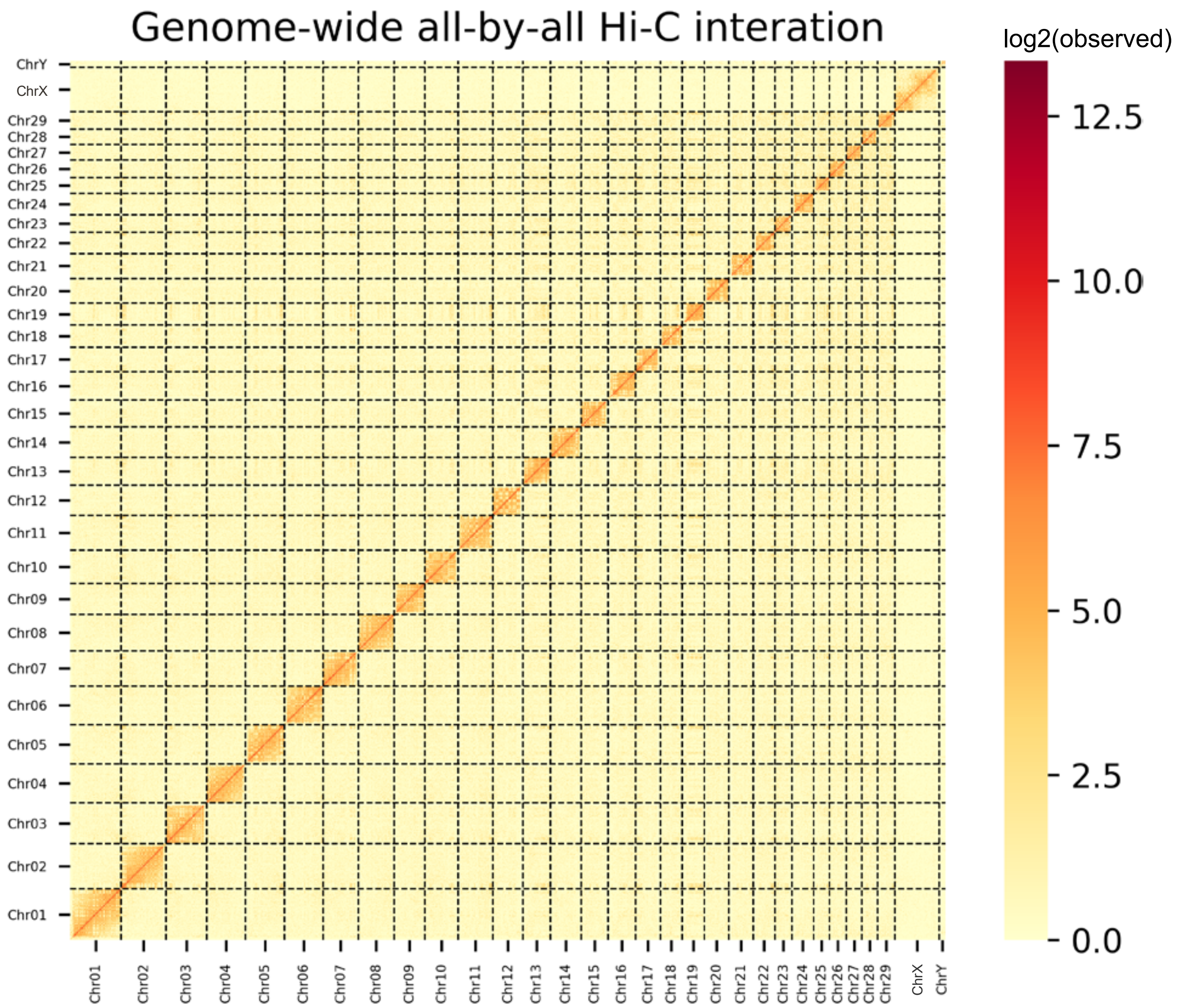

Supplementary Fig. 2 Hi-C heatmap for the chromosomes of the T2T-goat1.0 assembly. All chromosomes were assessed with Hi-C heatmap.

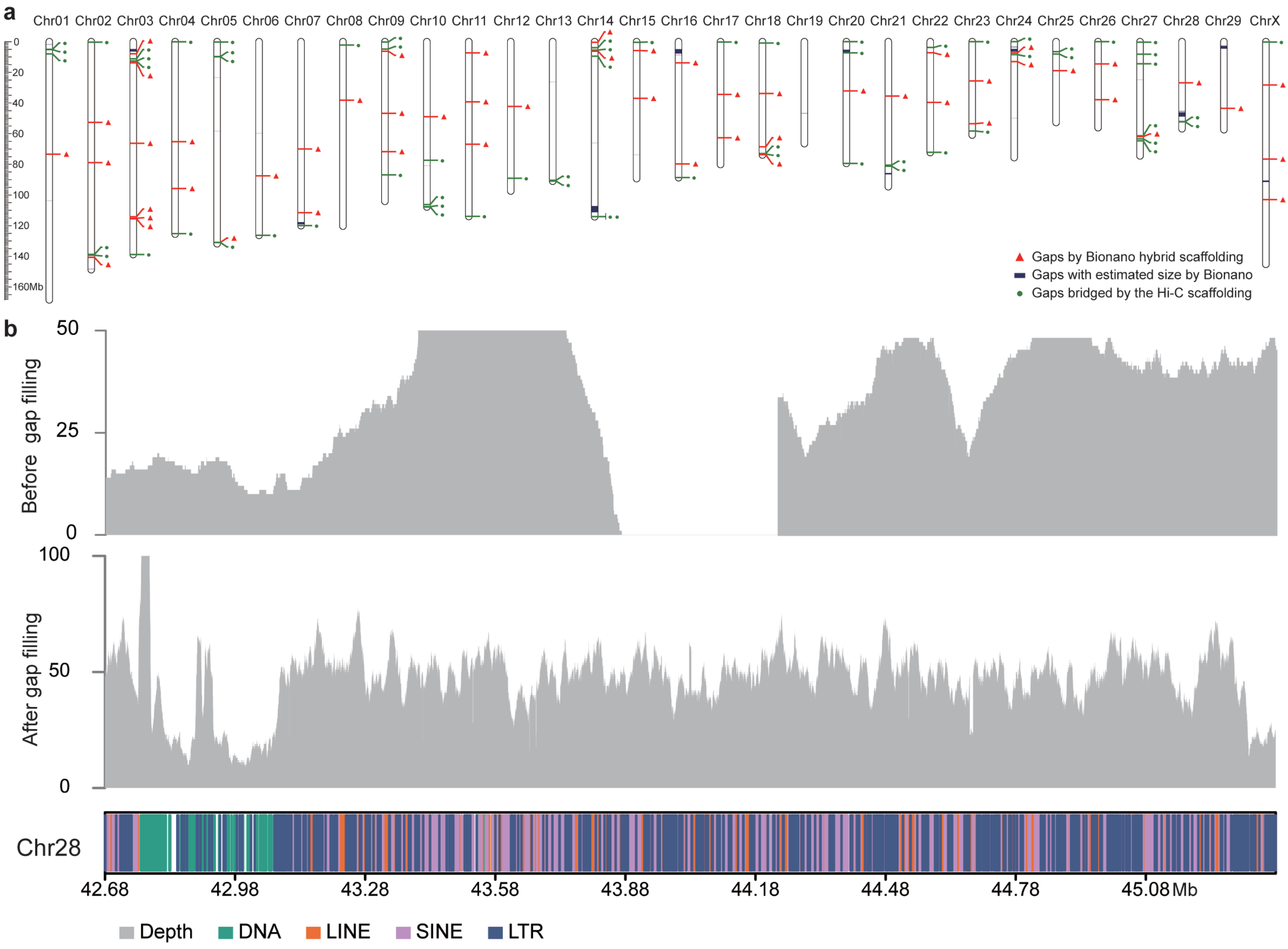

Supplementary Fig. 3 Gap filling and validation. a, Gap distribution across the chromosomes. Red triangles indicate the gaps generated based on the conflicting sites between contigs and Bionano optical maps during Bionano hybrid scaffolding. The blue bars indicate the gaps with an estimated size between the contigs in Bionano scaffolding. The green stars indicate the gaps bridged by the Hi-C scaffolding. **b,** A gap on chromosome 28, where the repetitive sequences are enriched, was filled and the gap-filled region was validated via the alignment of HiFi long reads.

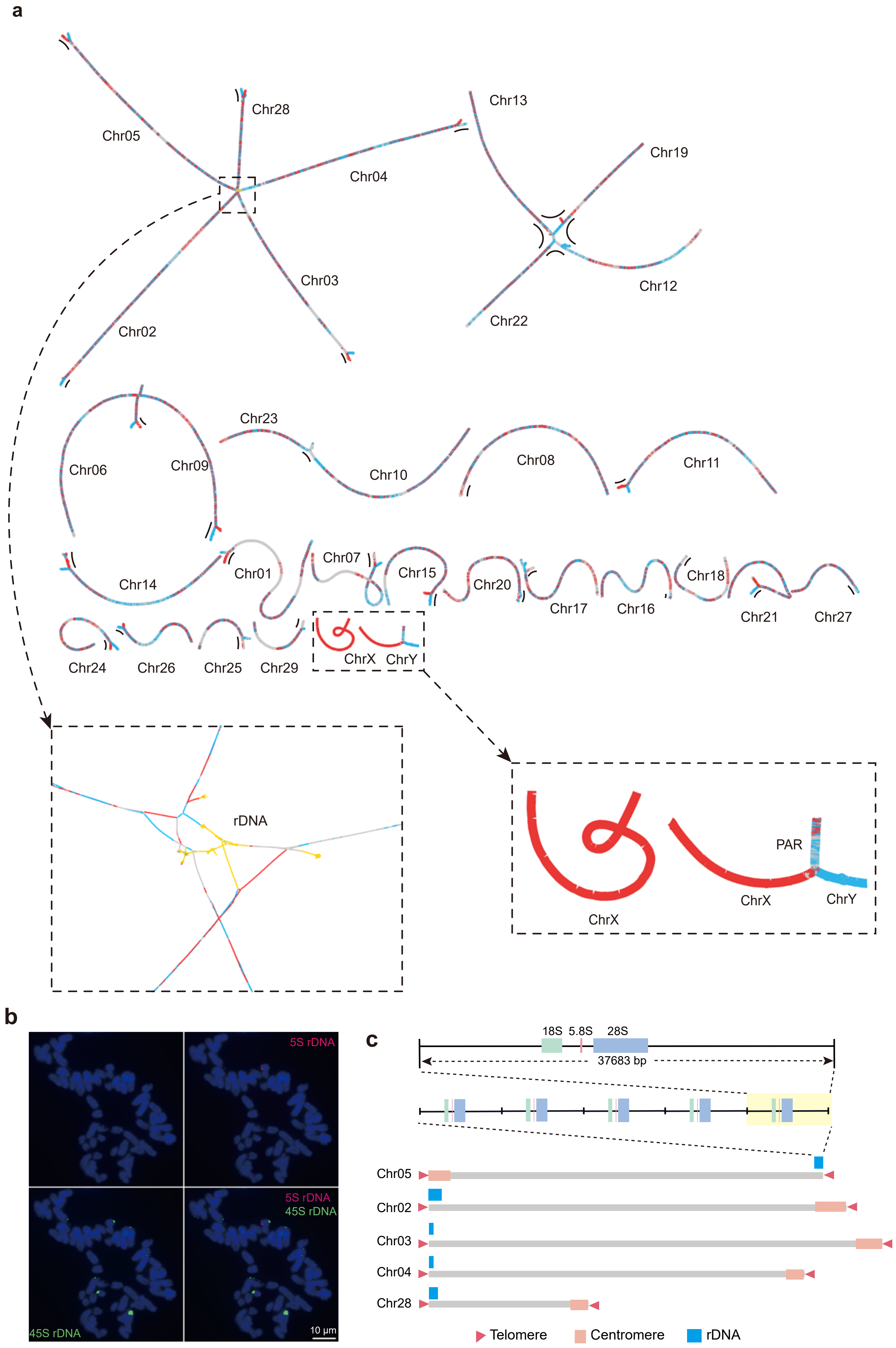

Supplementary Fig. 4 Assembly graph string and rDNA on the chromosomes. a, Assembly graph strings for the chromosomes. The chromosomes are colored in red for maternal *k*-mers, in blue for paternal *k*-mers, and in gray for no parental specific *k*-mers. The centromeric regions of the four chromosomes, Chr12, Chr13, Chr19 and Chr22, are entangled, and further enlarged and shown with centromeric regions highlighted in Fig. 1b. Another tangle related to 45S rDNA repetitive sequences (yellow lines in bottom left box) involve five chromosomes, Chr02, Chr03 Chr04, Chr05, and Chr28. The pseudoautosomal region (PAR) is shared between ChrX (two fragments in red) and ChrY (in blue), which are in the bottom right box. **b,** FISH images for the probes of 45S rDNA on five chromosomes (green) and 5S rDNA on Chr28 (red). **c,** 45S rDNA repetitive sequences that comprise of 18S, 5.8S, and 28S are located on the distal ends of Chr2, Chr3, Chr4, Chr5, and Chr28.

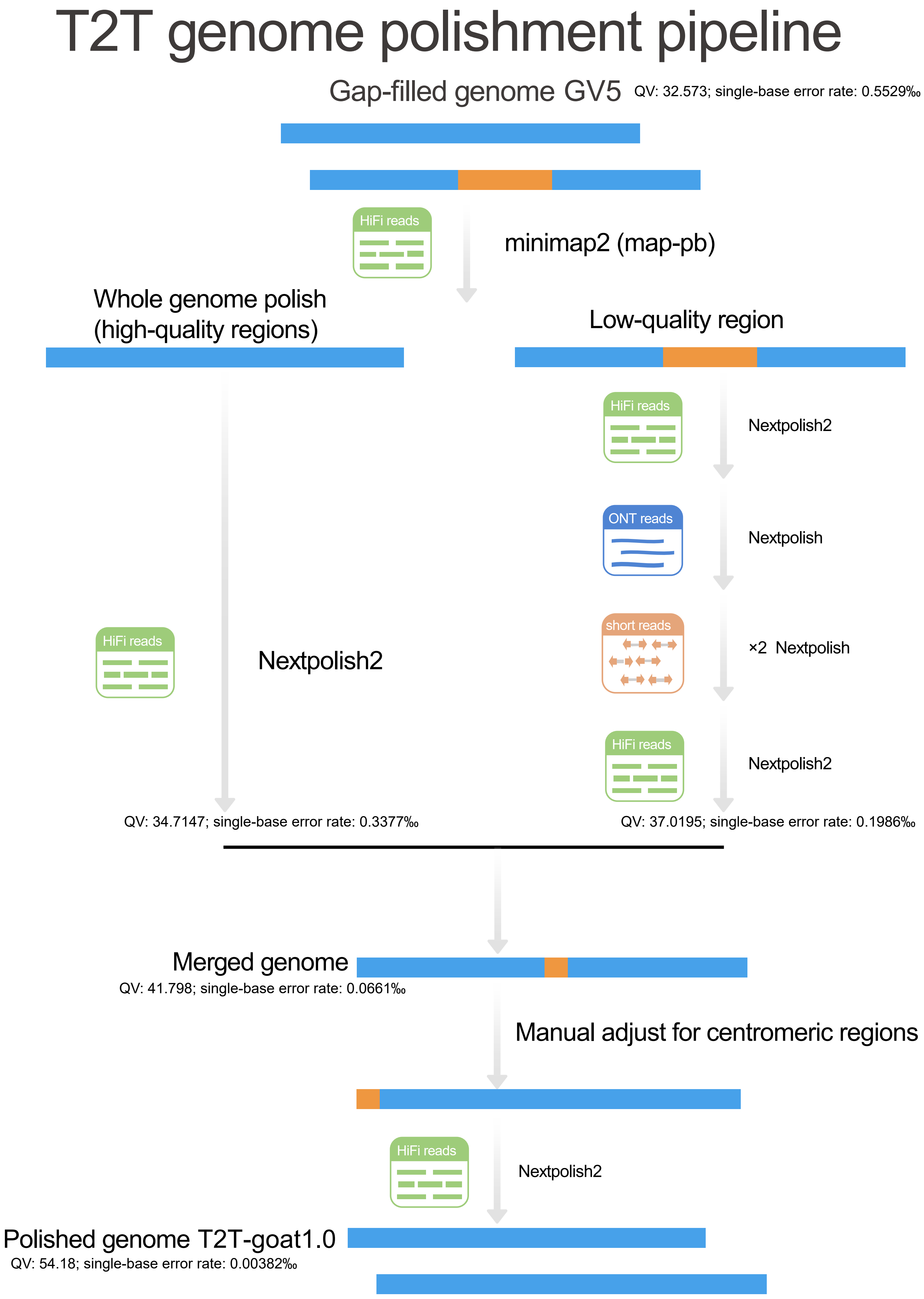

Supplementary Fig. 5 Polish pipeline for T2T-goat1.0. The genome assembly GV5 was polished with a pipeline of polishing based on HiFi, MGI, and ONT reads, by using Nextpolish and Nextpolish2 tools. The QV and single-base error rate are shown for the key steps.

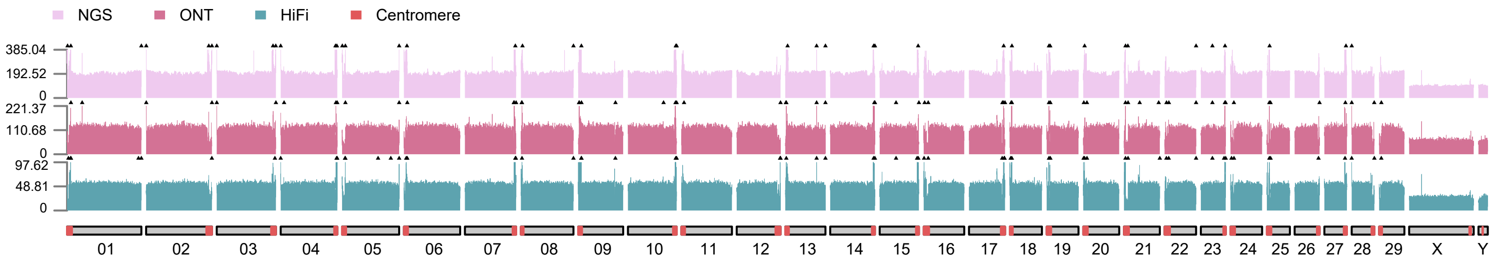

Supplementary Fig. 6 Coverage and issues of 31 chromosomes in T2T-goat1.0. Coverages of NGS (purple), ONT (red), and HiFi (blue) reads are shown for T2T-goat1.0. The low-coverage (a drop due to <0.5 folds of the average coverage) and high-coverage (an increase due to >2 folds of the average coverage) regions are highlighted for the potential assembly issues. The centromeres are highlighted in red for the chromosomal bars (gray) in the bottom.

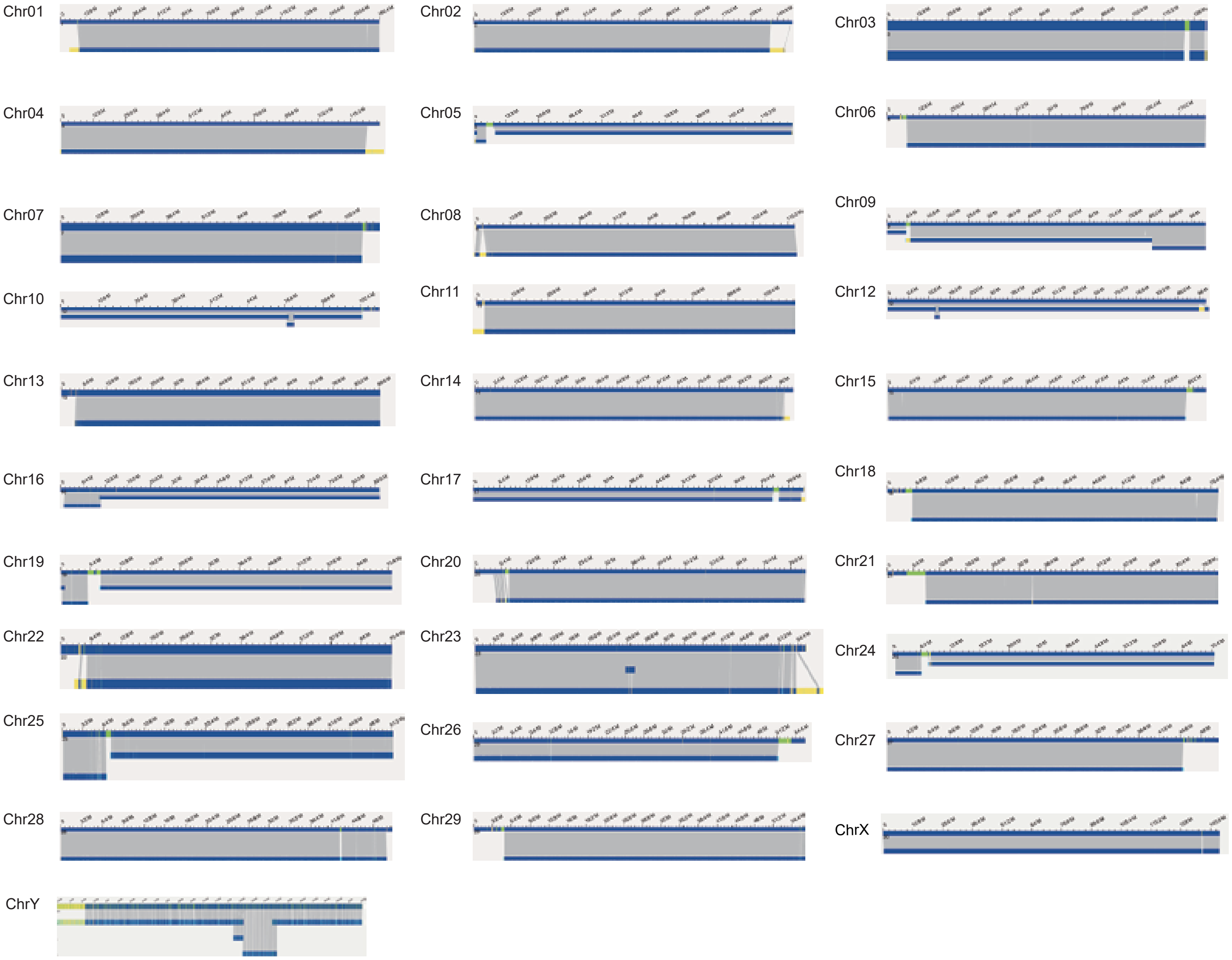

Supplementary Fig. 7 Bionano alignments against *in-silico* maps of 31 chromosomes in T2T-goat1.0. The top bars represent the T2T-goat1.0 chromosomes, and Bionano optical maps were aligned below. Blue color indicates the regions with consistencies and collinearity is represented by grey lines between the optical maps of T2T-goat1.0 chromosomes and Bionano hybrid assembly. Meanwhile, yellow regions represent breaks in collinearity between T2T-goat1.0 chromosomes and Bionano hybrid assembly, while green regions without enzyme labeling sites are the stretches of unique sequences in T2T-goat1.0 without collinearity against Bionano maps below. Light blue regions in Bionano maps for hybrid assembly below indicate the stretches of unique sequences without enzyme labeling sites. The centromeric regions are enriched with repeats, and lack of enzyme labeling sites for Bionano. Therefore, green and yellow regions are found mostly in the ends of the acrocentric chromosomes, which indicate the locations of centromeres. So are green and yellow regions in the pseudoautosomal region (PAR) of chromosome Y.

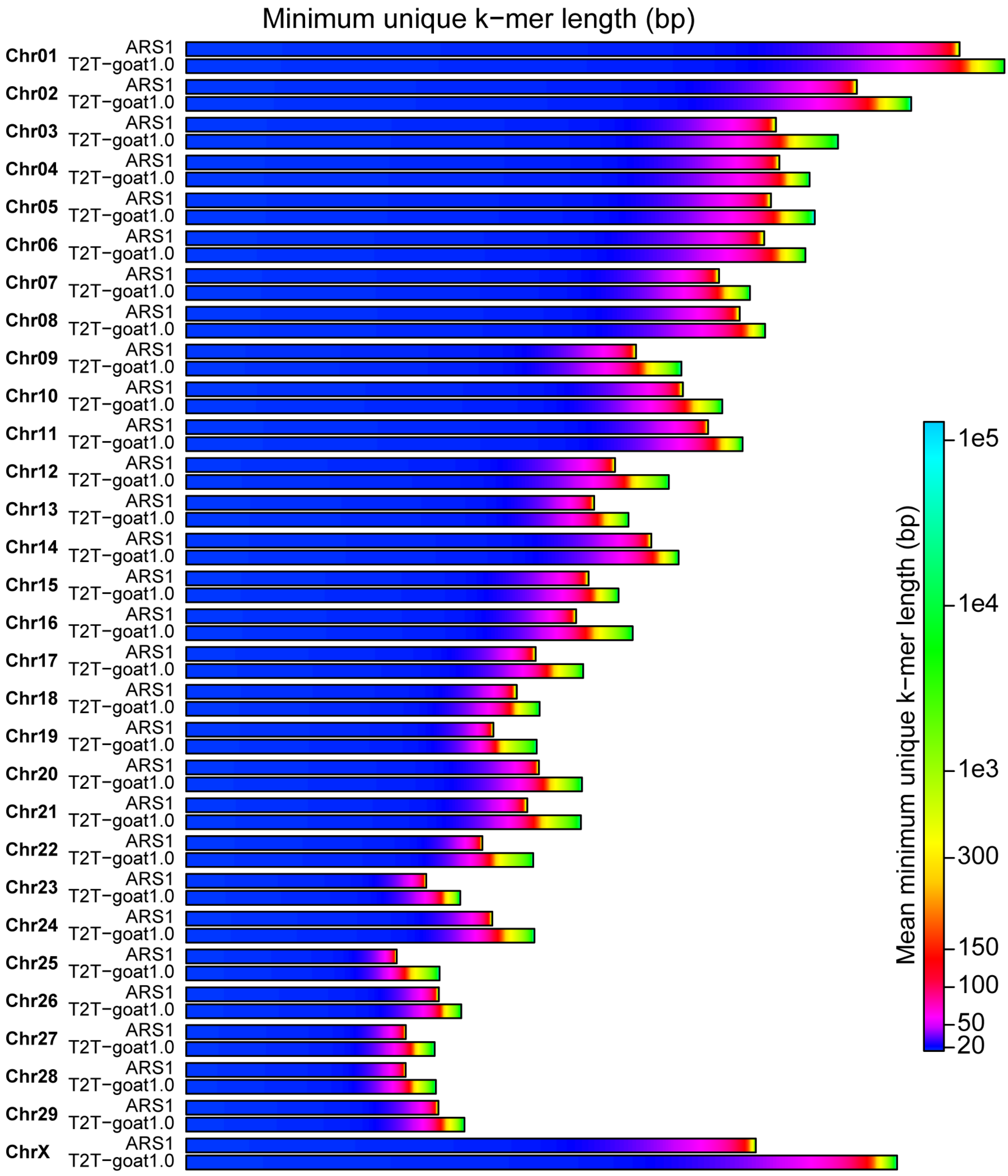

Supplementary Fig. 8 Length of minimum unique *k*-mers in T2T-goat1.0 compared to ARS1. Minimum unique *k*-mers (MUKs) were calculated in 100-kb windows for the chromosomes of both T2T-goat1.0 and ARS1, according to T2T Minimum Unique K-mer Analysis pipeline (https://github.com/msauria/T2T_MUK_Analysis). The more MUK values indicate more repetitive sequences in a 100-kb window.

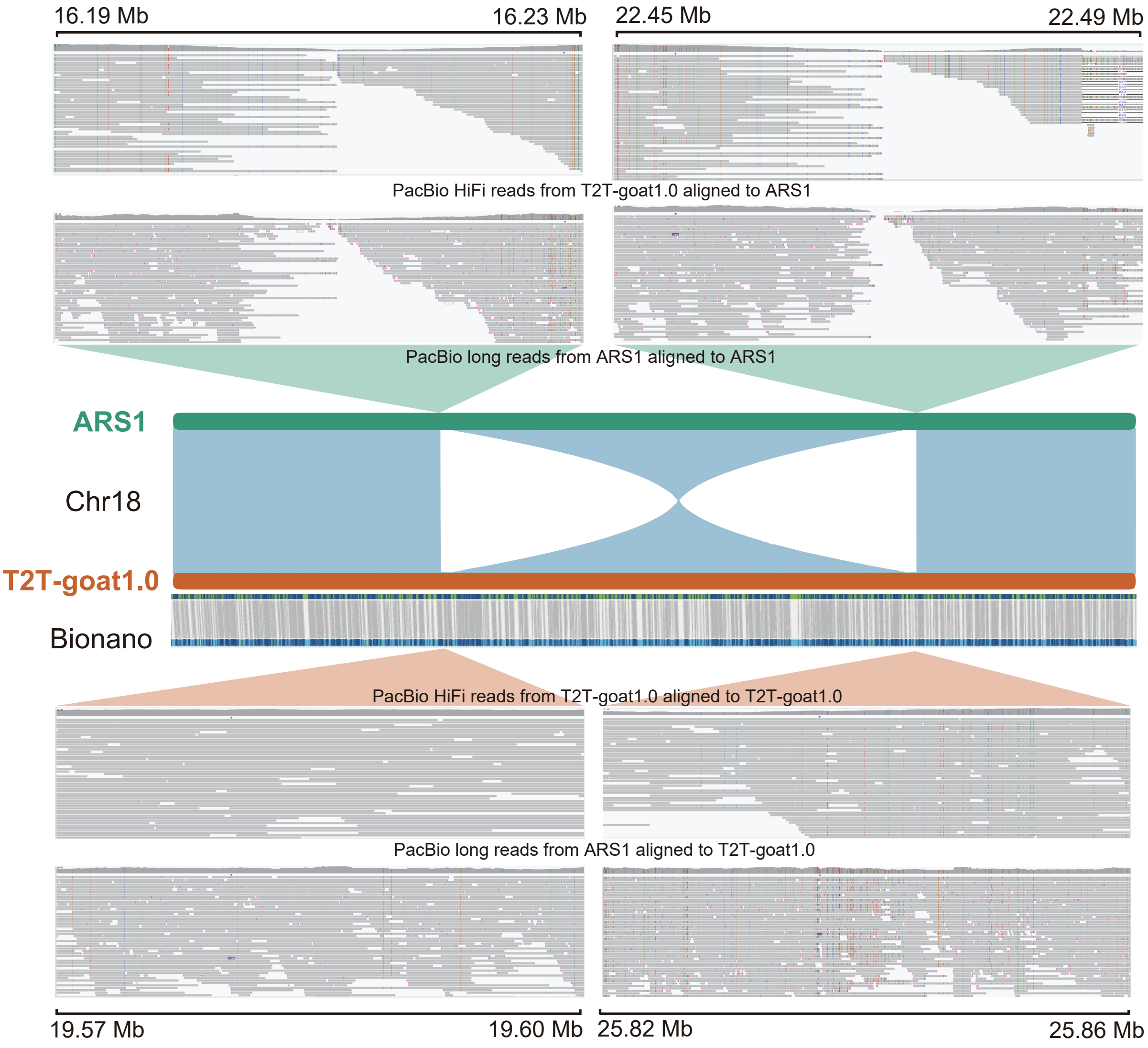

Supplementary Fig. 9 An inversion error on chromosome 18 of ARS1. The two junction sites of an inversion on chromosome 18 of ARS1 could not be covered by the PacBio reads sequenced for the T2T-goat1.0 and ARS1 individual (NCBI accession no. PRJNA340281), with the evidences of the clipped reads for alignments to ARS1 visualized with IGV. The corresponding sites in T2T-goat1.0 were assembled correctly with even coverage of long-read from both ARS1 and T2T-goat1.0, and the accurate assembly of this region is further supported by Bionano alignment.

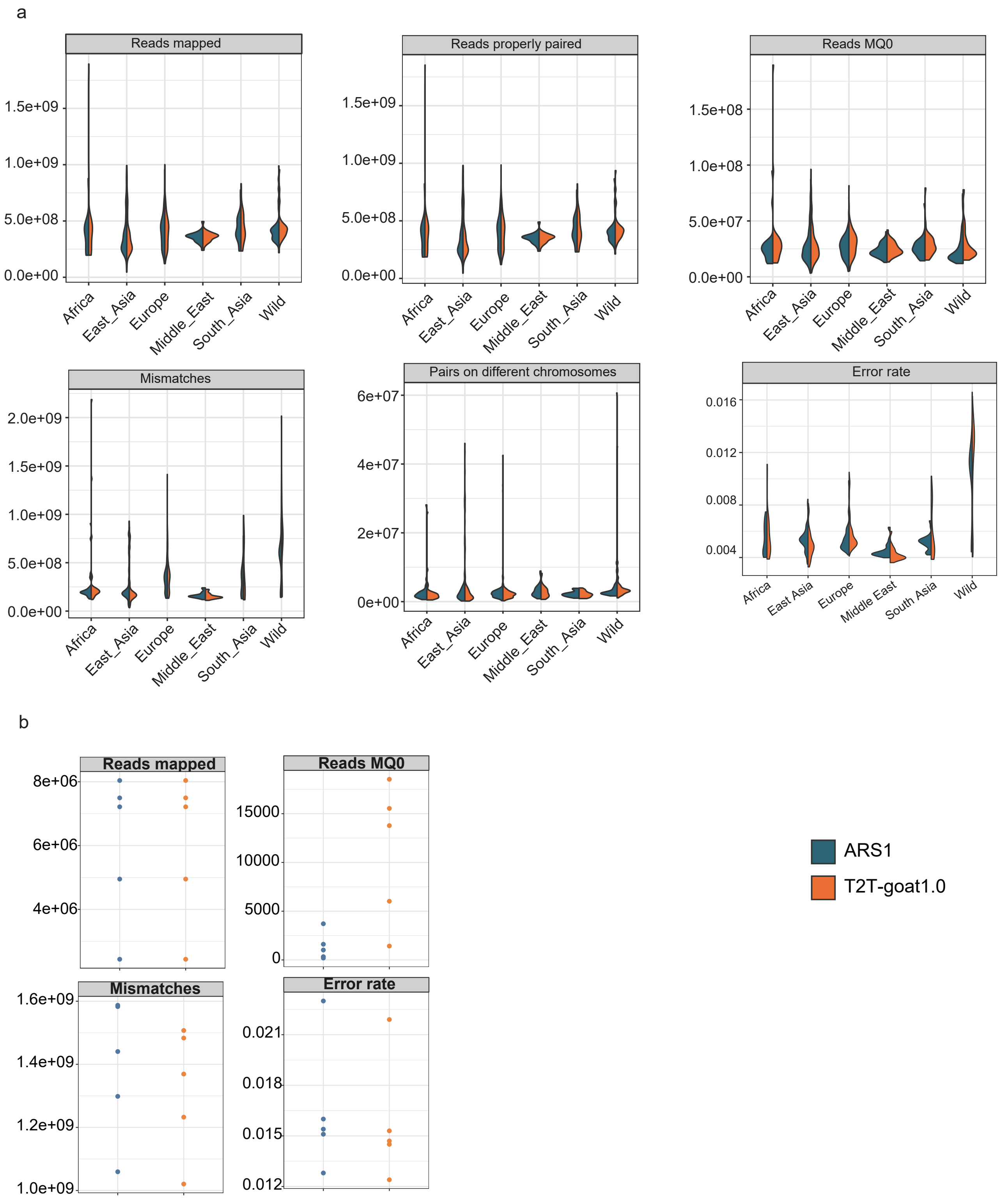

Supplementary Fig. 10 Summary of alignments for short and long reads in a comparison between T2T-goat1.0 and ARS1 as references. a, The short reads were derived from a total of 516 individuals from 5 geographic domestic goat populations (Africa, East Asia, Europe, the Middlte East, and South Aisa) and wild goats, and mapped reads, properly paired mapped reads, MQ0, mismatches, paired reads on different chromosomes, and error rates are compared in the five populations. **b,** T2T-goat1.0 and ARS1 as references are compared for alignments of long reads from five newly sequenced goats, including mapped reads, MQ0, mismatches, and error rates.

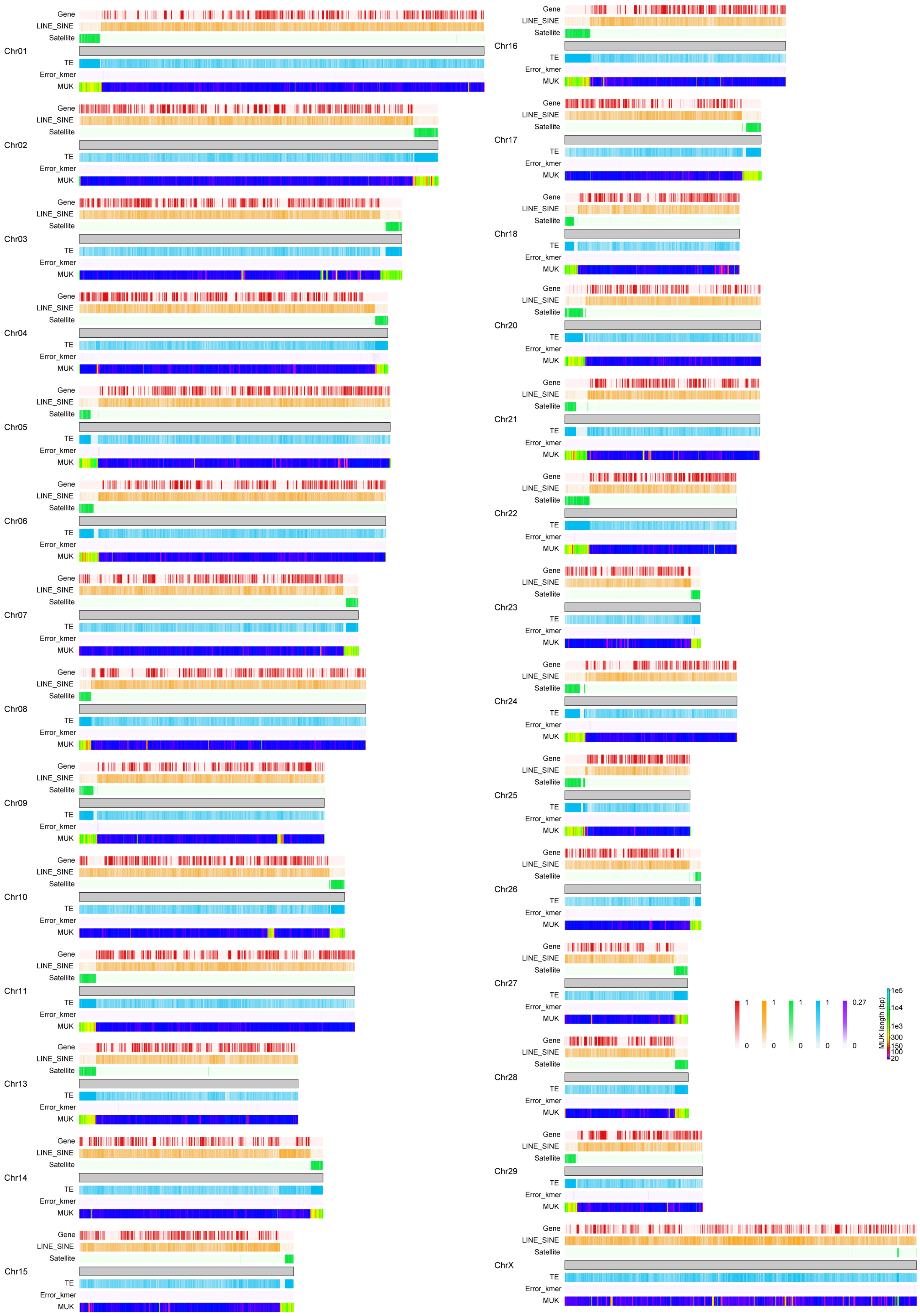

Supplementary Fig. 11 Distribution of genomic features across the chromosomes. Centromeric regions were predicted by RepeatMasker. The gene density, LINE and SINE (LINE-SINE), satellite, transposable element (TE), error *k*-mer (*k*=21), and minimum unique *k*-mer (MUK) are shown in 100-kb windows.

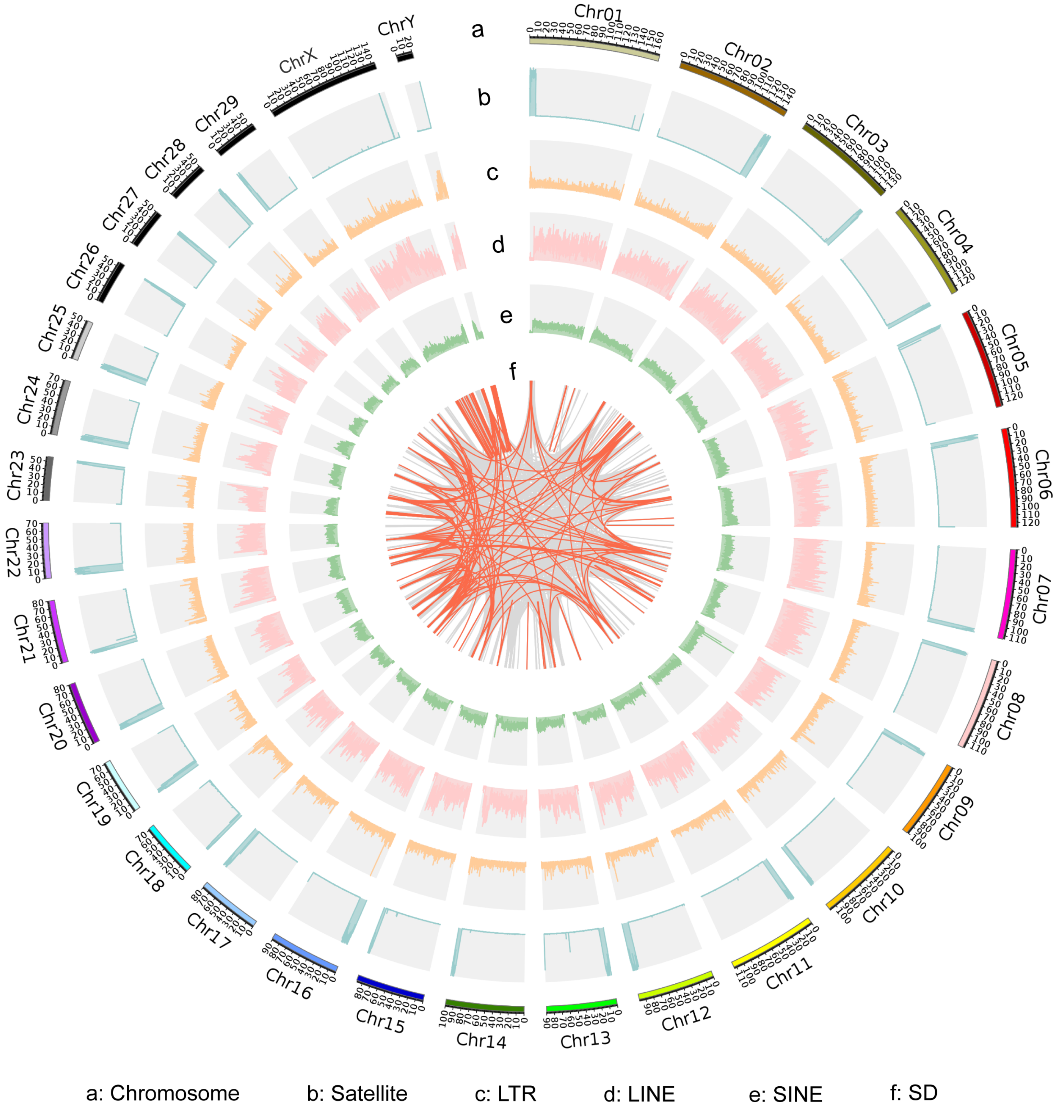

Supplementary Fig. 12 Repetitive sequences on T2T-goat1.0. The y-axis labels are given from the outer to the inner as follows: Chromosome (**a**), Satellite (**b**), LTR (**c**), LINE (**d**), and SINE (**e**). **f,** The red and gray lines in the inner circle represent segmental duplications (SDs) in the PURs and the whole T2T-goat1.0 genome respectively.

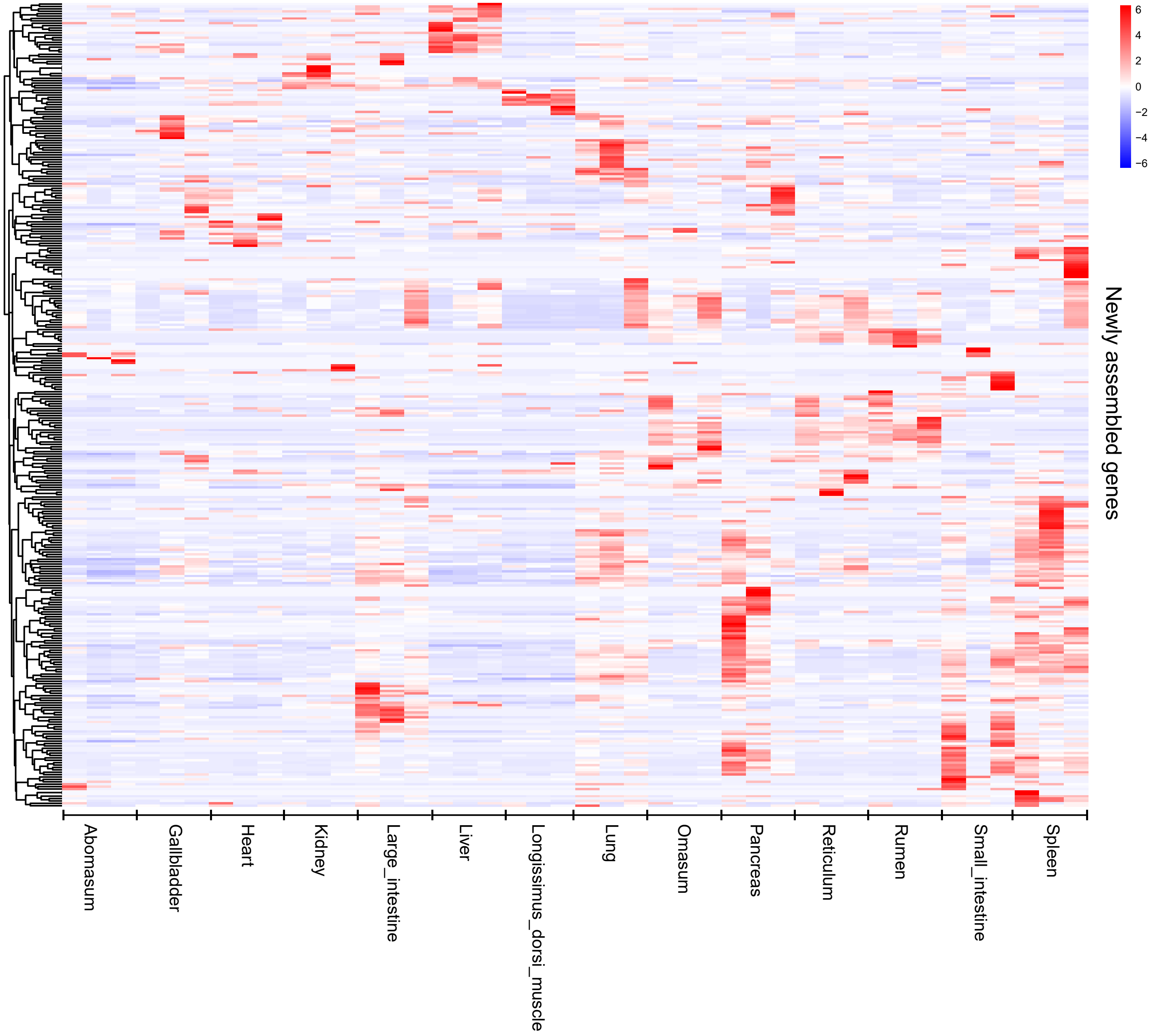

Supplementary Fig. 13 Expressions of newly assembled genes in T2T-goat1.0. Expression of newly assembled genes across 14 goat tissues.

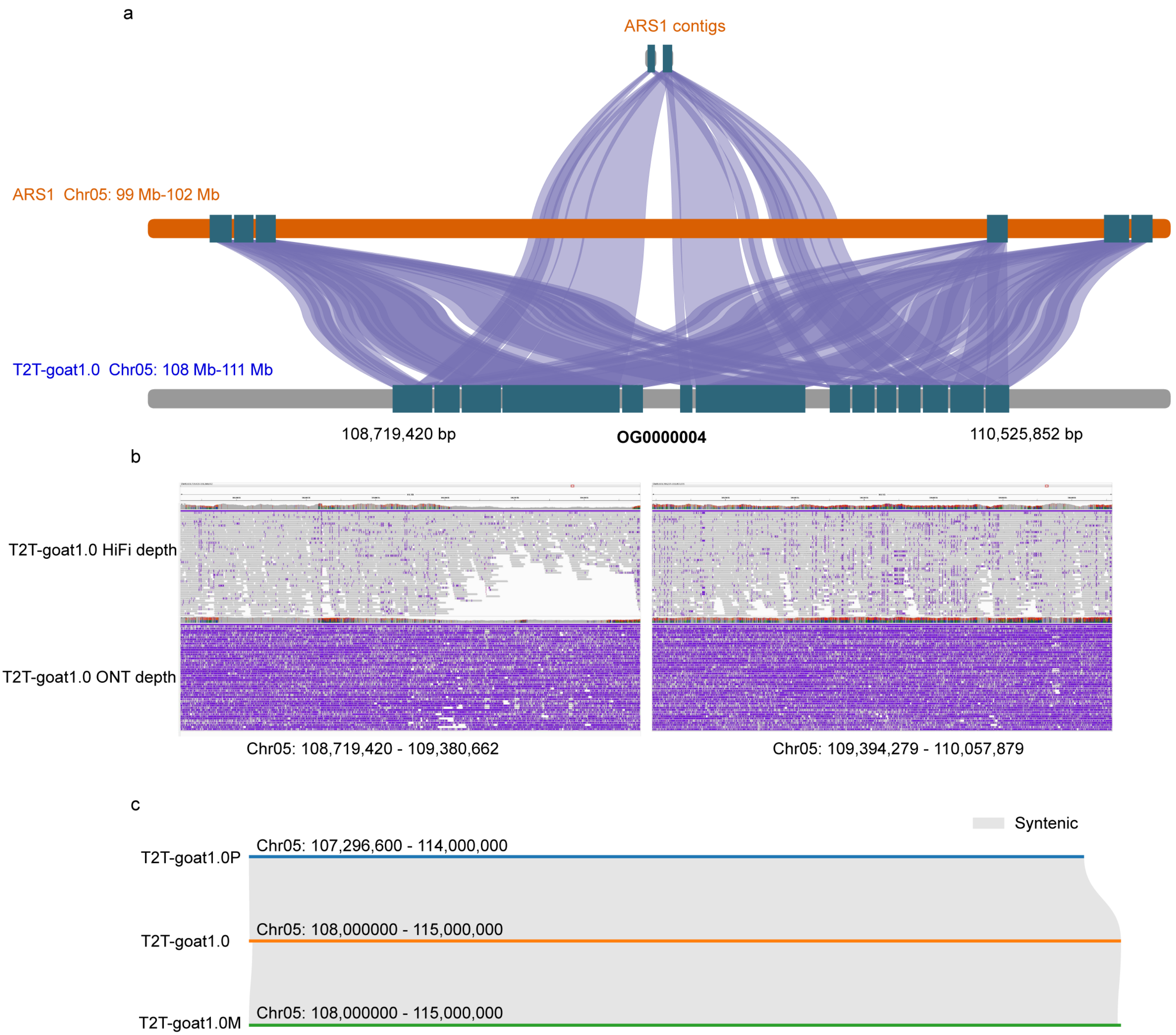

Supplementary Fig. 14 Assembly of the OG0000004 gene family genes in T2T-goat1.0 compared to that in ARS1. a, Collinearity of the region with tandem genes of the scavenger receptor activity (OG0000004) family is shown between T2T-goat1.0 and ARS1. The 15 genes in this gene family were assembled in a tandem way on Chr05 of T2T-goat1.0, but only 8 genes on Chr05 and unplaced contigs of ARS1. **b,** The assembly of the region containing OG0000004 is examined for alignments of HiFi and ONT reads against T2T-goat1.0. **c,** The collinearity between T2T-goat1.0P and T2T-goat1.0M confirmed the accurate assembly of this region containing OG0000004 on Chr05, rather than the assembly errors due to the heterozygosity between the haplotype genome assemblies.

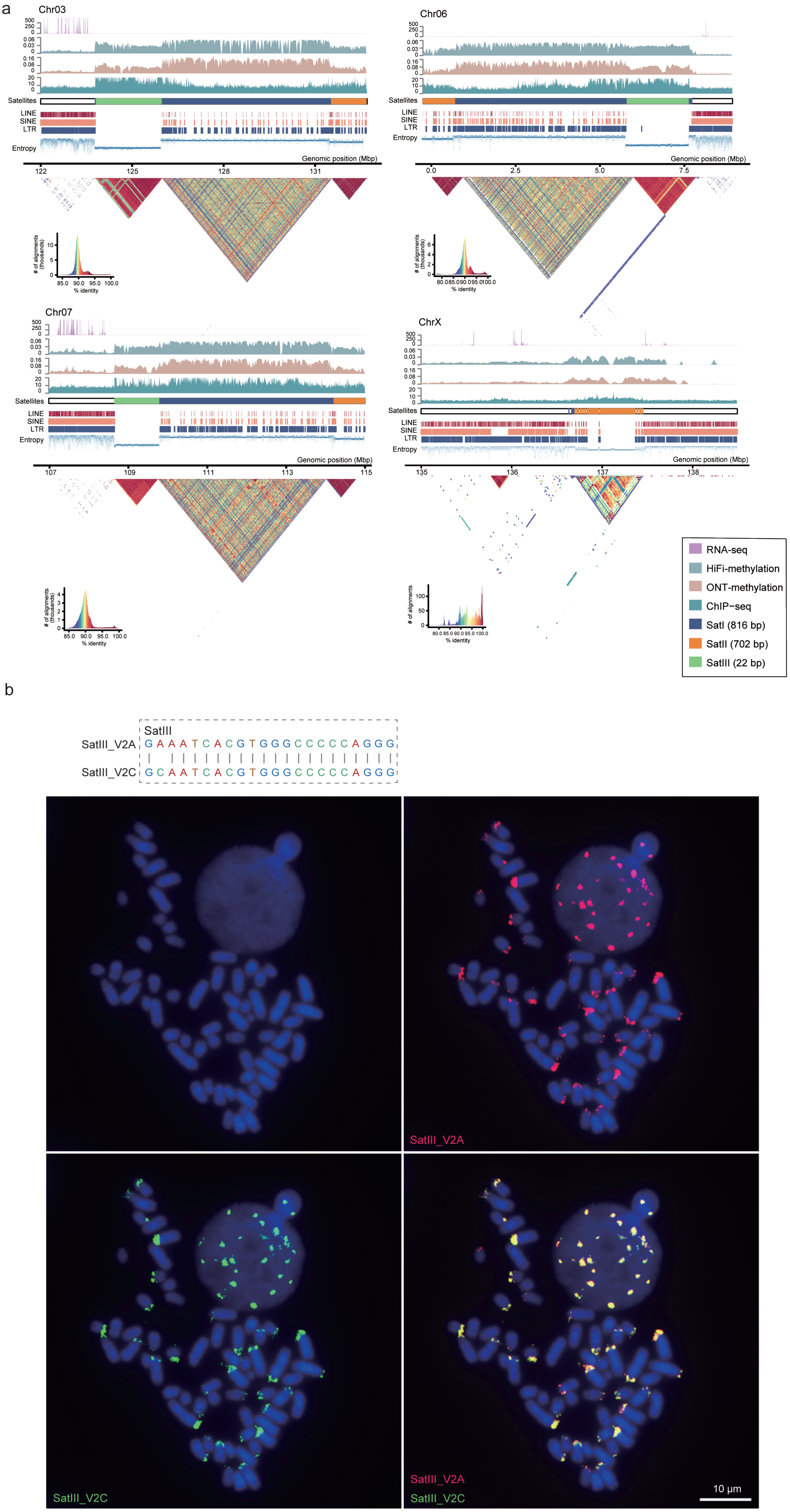

Supplementary Fig. 15 Centromeric regions and their compositions of the repetitive sequences in T2T-goat1.0. a, Features in centromeric regions of four chromosomes (Chr03, Chr06, Chr07, and ChrX). The labels and colors are the same to those in Fig. 3a. **b,** Two variants (SatIII-V2A and SatIII-V2C) of SatIII (22 bp) with one nucleotide variation (top) and FISH images with their probes (red and green), with experimental replicates (n = 5).

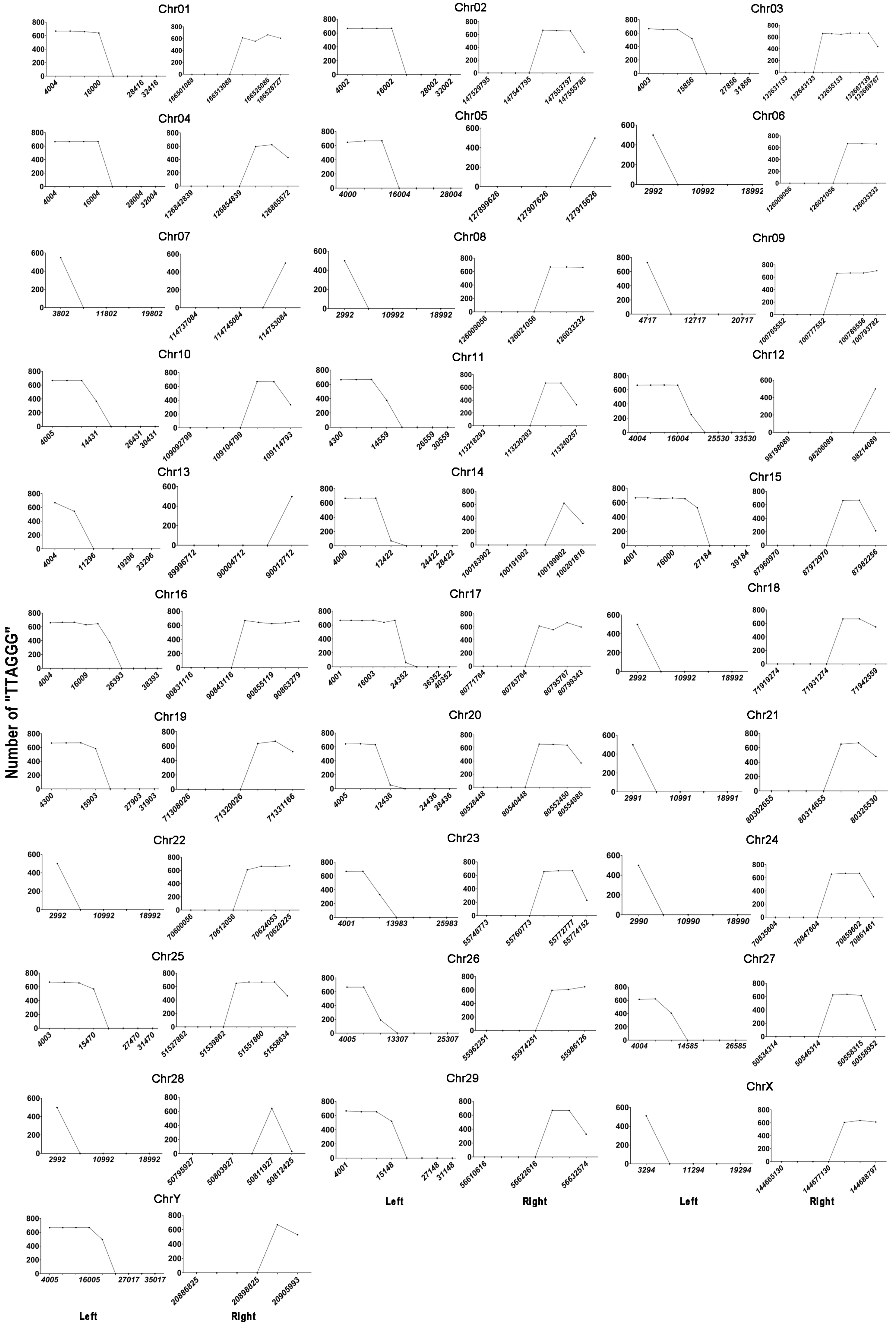

Supplementary Fig. 16 Telomeric lengths on the chromosomal ends of T2T-goat1.0.

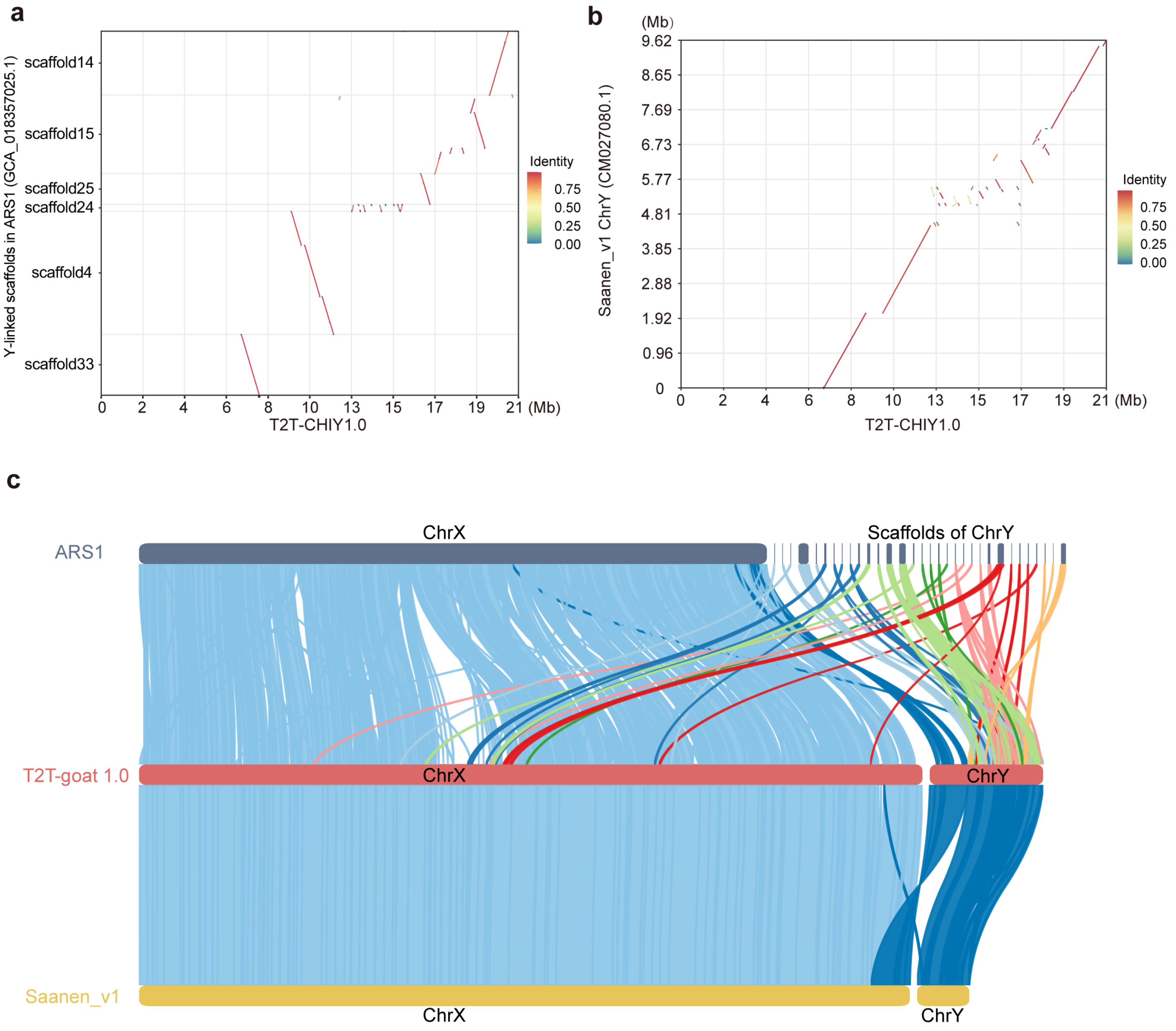

Supplementary Fig. 17 Syntenic regions between the X and Y chromosomes among the three assemblies of T2T-goat1.0, ARS1 and Saanen_v1. a, Collinearity of the Y chromosomes or scaffolds of ARS1 and T2T-CHIY1.0. **b,** Collinearity of the Y chromosomes of Saanen_v1.0 and T2T-CHIY1.0. **c,** The syntenic regions are observed between chromosomes X (ChrX) and Y (ChrY). The chromosomes (or scaffolds) X and Y in ARS1 and Saanen_v1 both show the high collinearity with that in T2T-goat1.0. Lines for collinearity are shown in random colors between ChrY scaffold of ARS1 and ChrX and ChrY of T2T-goat1.0.

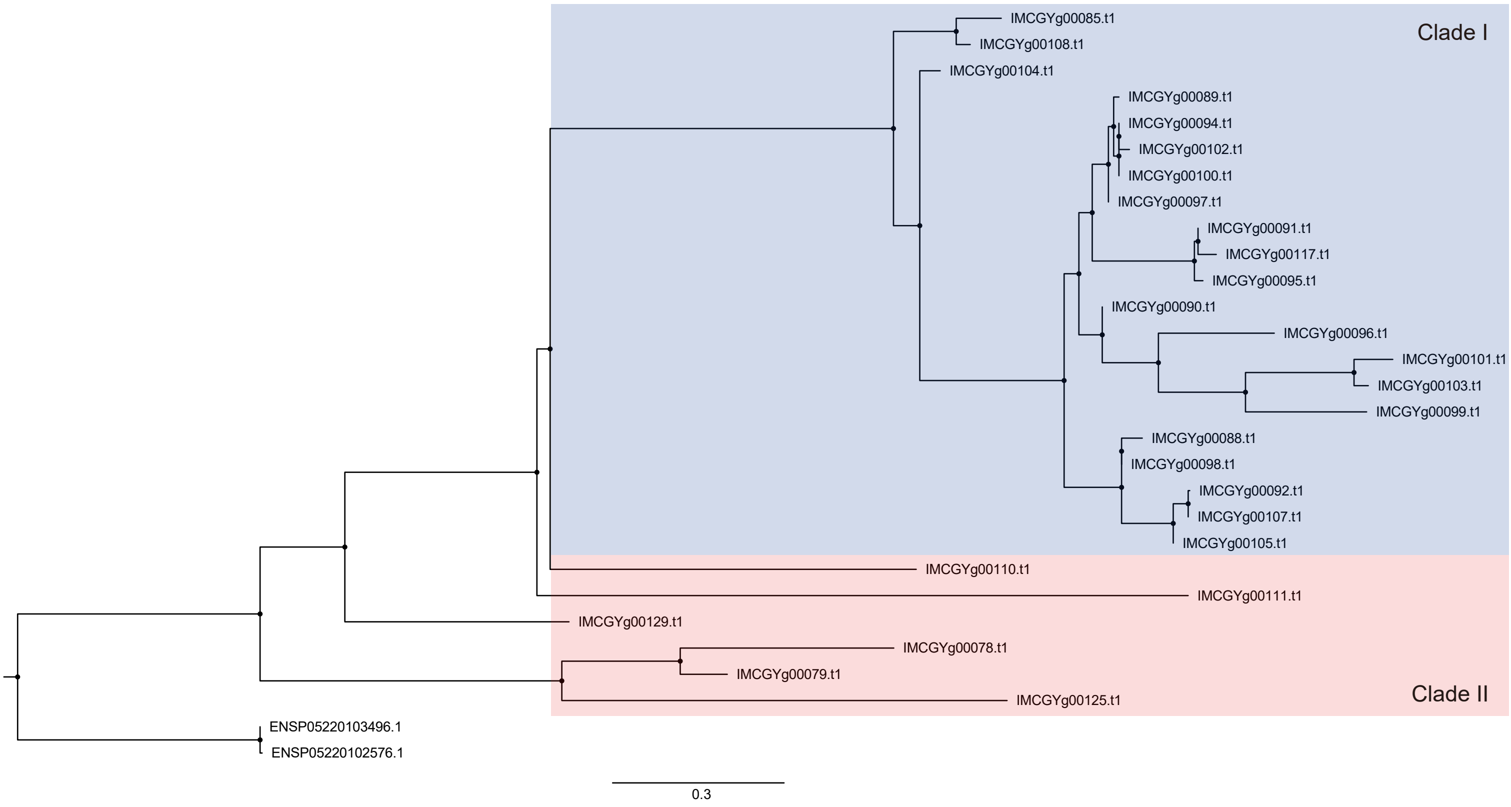

Supplementary Fig. 18 Phylogenetic tree for *TSPY* genes. Two human Y chromosomal *TSPY* genes (ENSP05220103496.1 and ENSP05220102576.1) were selected as the outgroup. The amino acid sequences were used to construct the maximum-likelihood tree under the HKY nucleotide substitution mode, which grouped *TSPY* genes into two clades, Clade I and Clade II.

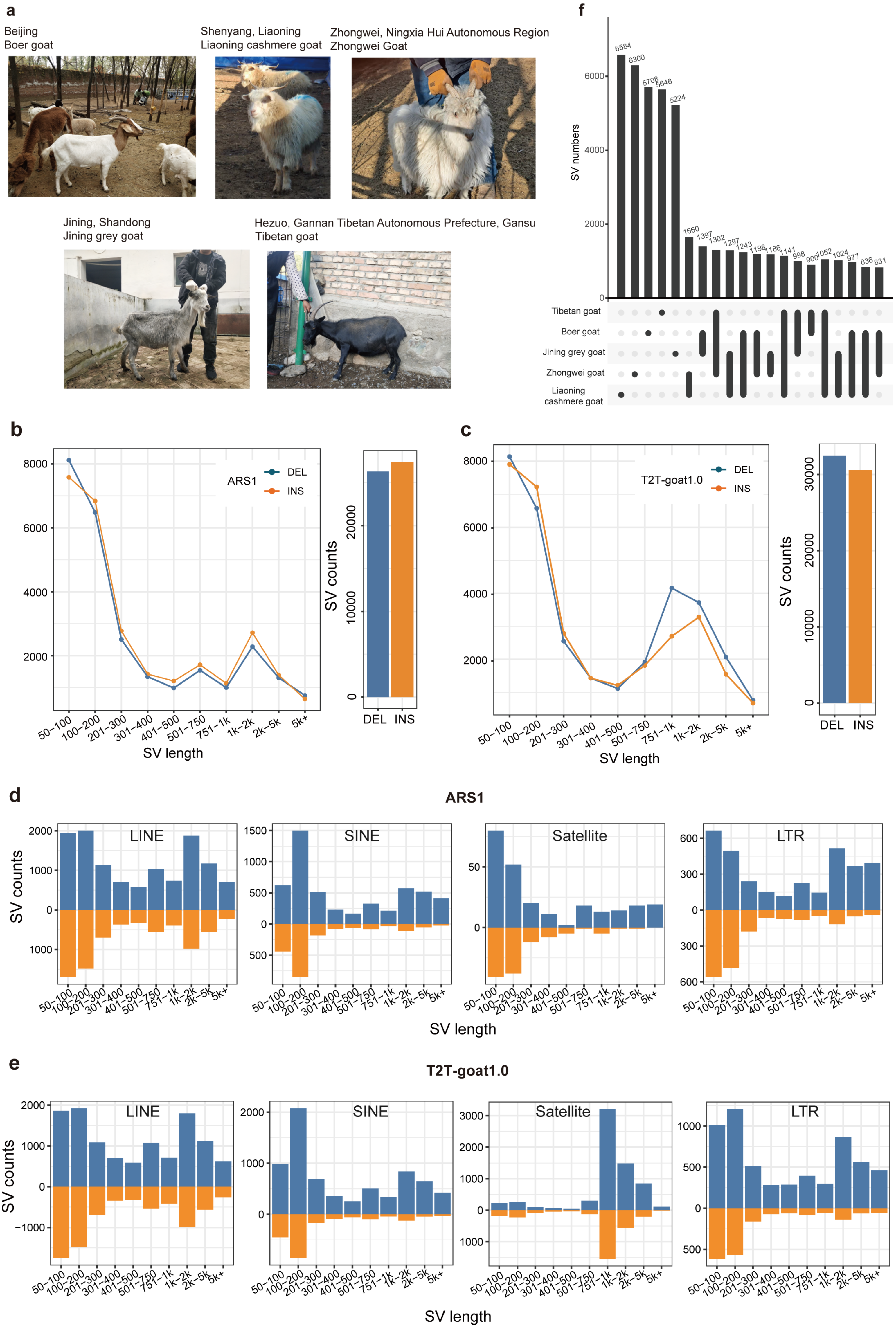

Supplementary Fig. 19 SV calling based on the PacBio long reads of five representative goats. a, Five goats from China (Boer for BEG, Jining gray goat for JNGG, Liaoning cashmere goat for LNCG, Tibetan goat for TG, and Zhongwei goat for ZWG) were selected to sequence PacBio reads. T2T-goat1.0 and ARS1 as references are compared for performances of calling SVs based on long reads of five goats. DEL, deletion; INS, insertion. Various lengths of DEL and INS are counted in a comparison of ARS1 (**b**) and T2T-goat1.0 (**c**). Various lengths of DEL and INS are counted in the repetitive sequences of LINE, SINE, Satellite, and LTR in a comparison of ARS1 (**d**) and T2T-goat1.0 (**e**). **f,** SVs uniquely identified in the five representative goats.

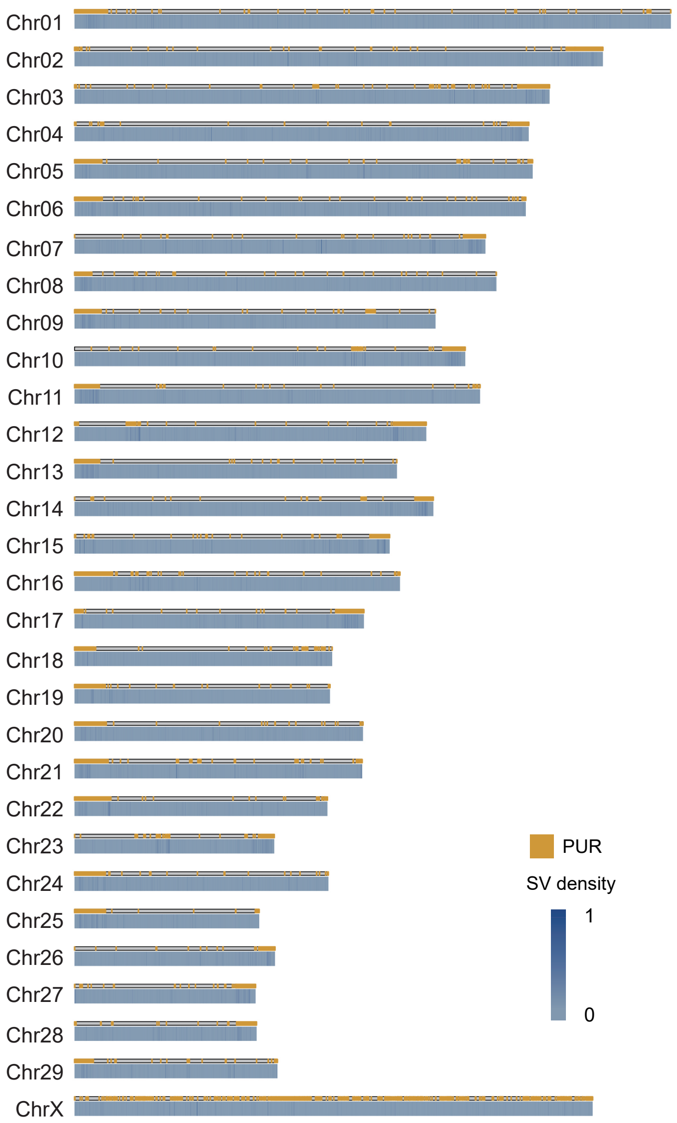

Supplementary Fig. 20 SVs based PacBio reads with T2T-goat1.0 as a reference. Density of SVs called from PacBio reads of the five representative goats with T2T-goat1.0 as a reference, in 10-kb windows. PURs are highlighted in yellow.

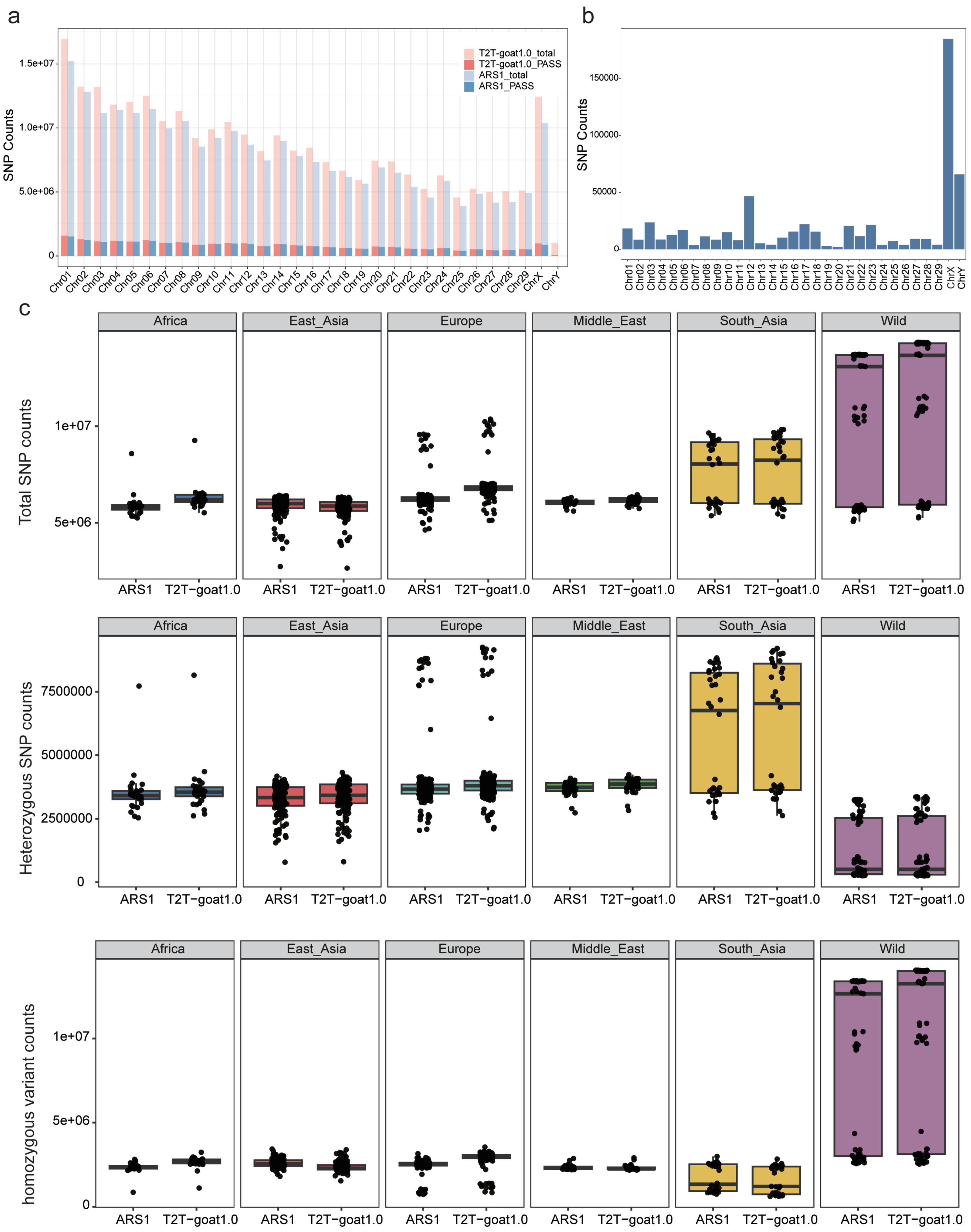

Supplementary Fig. 21 SNPs based on short reads in a comparison between ARS1 and T2T-goat1.0 as references. a, SNPs are compared across chromosomes between ARS1 and T2T-goat1.0. **b,** SNPs in the PURs are compared across chromosomes. **c,** All SNPs, heterozygous and homozygous SNPs are shown for the six geographic populations.

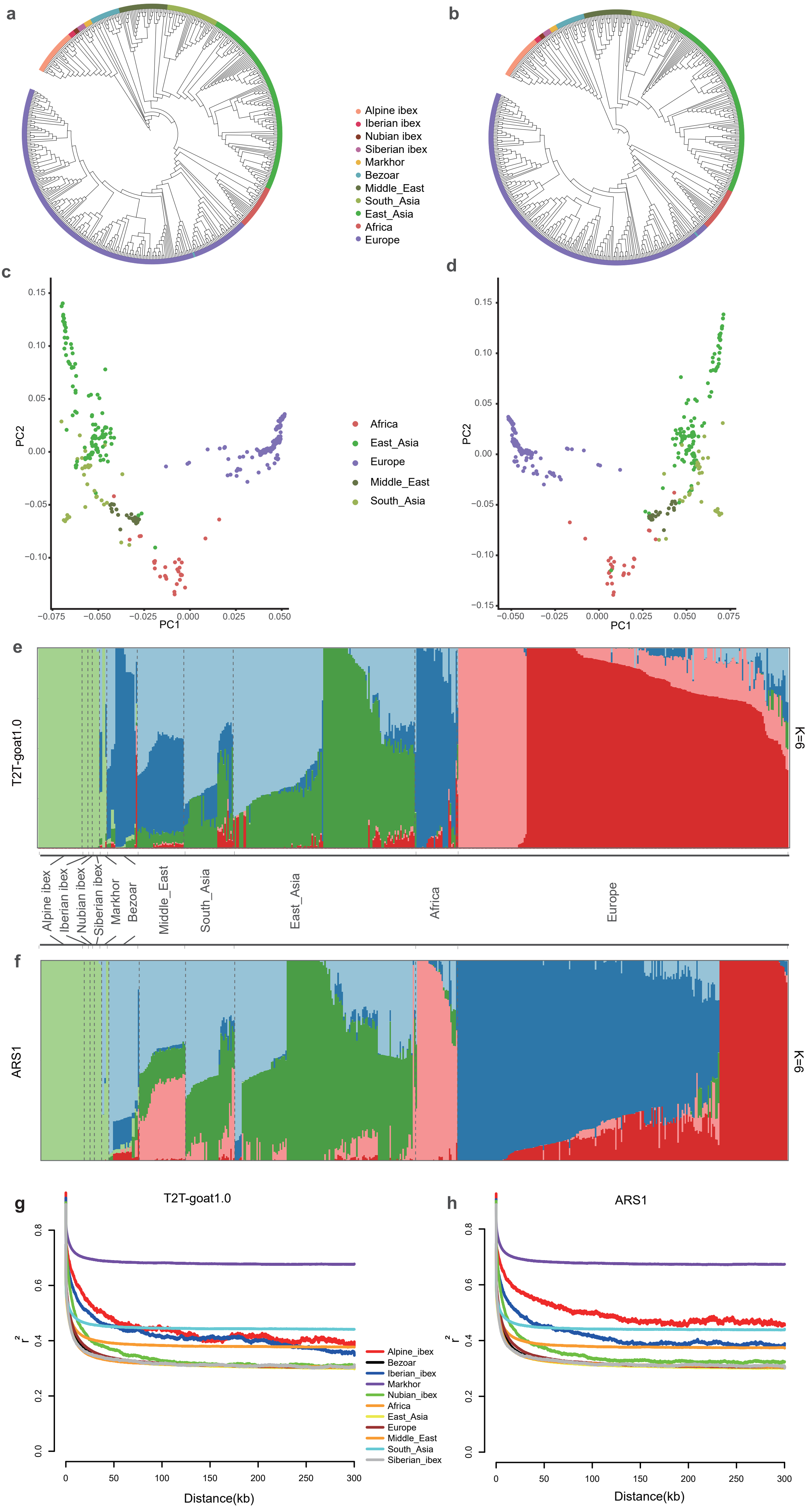

Supplementary Fig. 22 Population structure inferred with ARS1 and T2T-goat1.0 as references. The neighbor-joining tree of wild and domestic goats (**a,** T2T-goat1.0 as a reference **b,** ARS1 as a reference) and PCA of five geographic domestic goat populations (**c,** T2T-goat1.0 as a reference; **d,** ARS1 as a reference) were generated based on LD-pruned SNPs. Population genetic structure analysis (k=6) based on SNPs with T2T-goat1.0 (**e**) and ARS1 (**f**) as references showed the similar populational structure. Linkage disequilibrium (LD, r^2) based on SNPs showed a difference of Alpine ibex (red color) between T2T-goat1.0 (**g**) and ARS1 (**h**) as references.

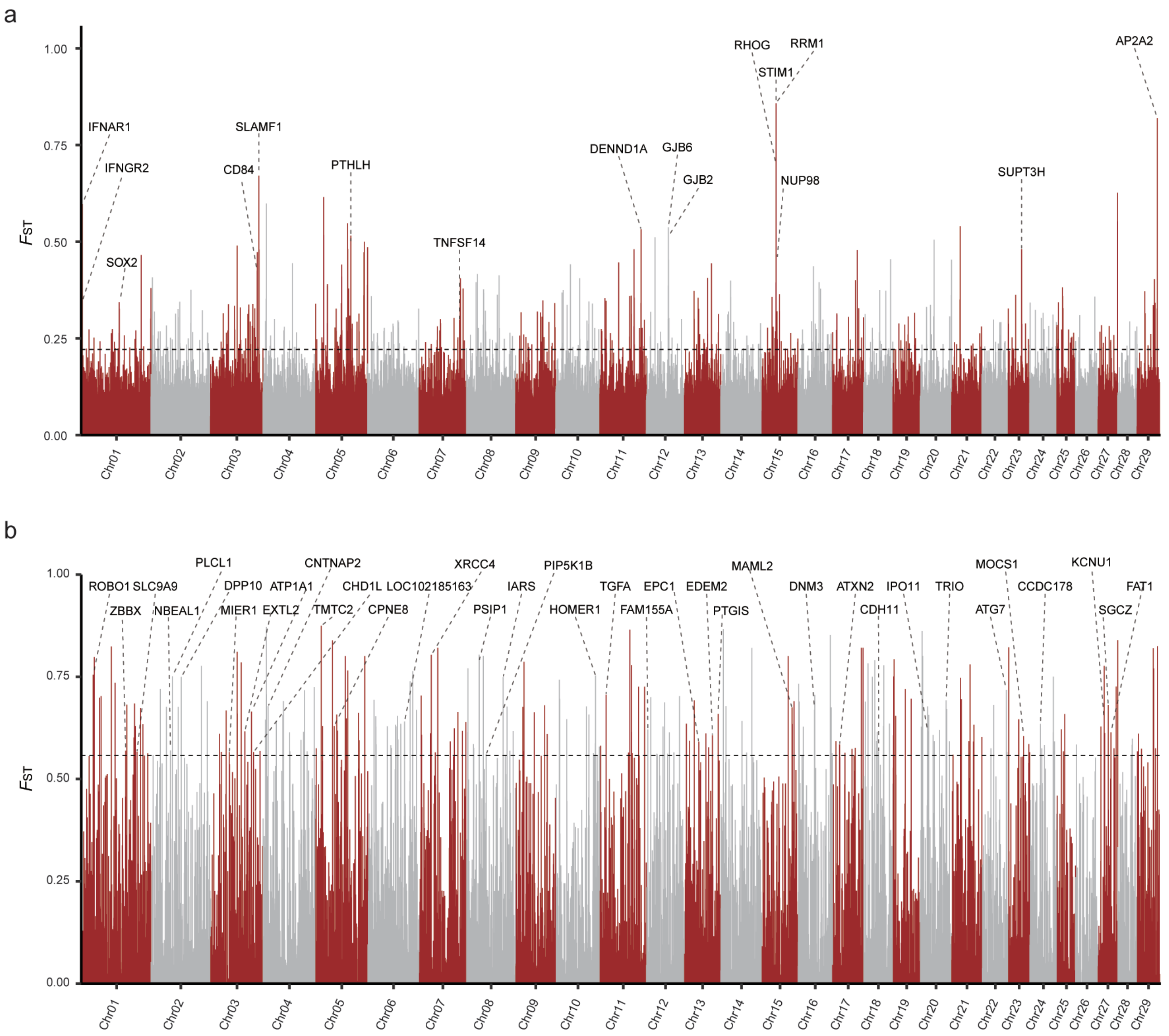

Supplementary Fig. 23 Manhattan plot of the genomic regions under selection associated with domestication using ARS1 as the reference genome. A, Selective signals between bezoars and domestic goats are determined based on SNPs using the top 1% F_{ST} (horizontal dash line). **b,** Selective signals between bezoars and domestic goats based on SVs are determined using the top 1% F_{ST} values (horizontal dash line). Gene symbols are shown if the genes are identified by using both ARS1 and T2T-goat1.0 as references.

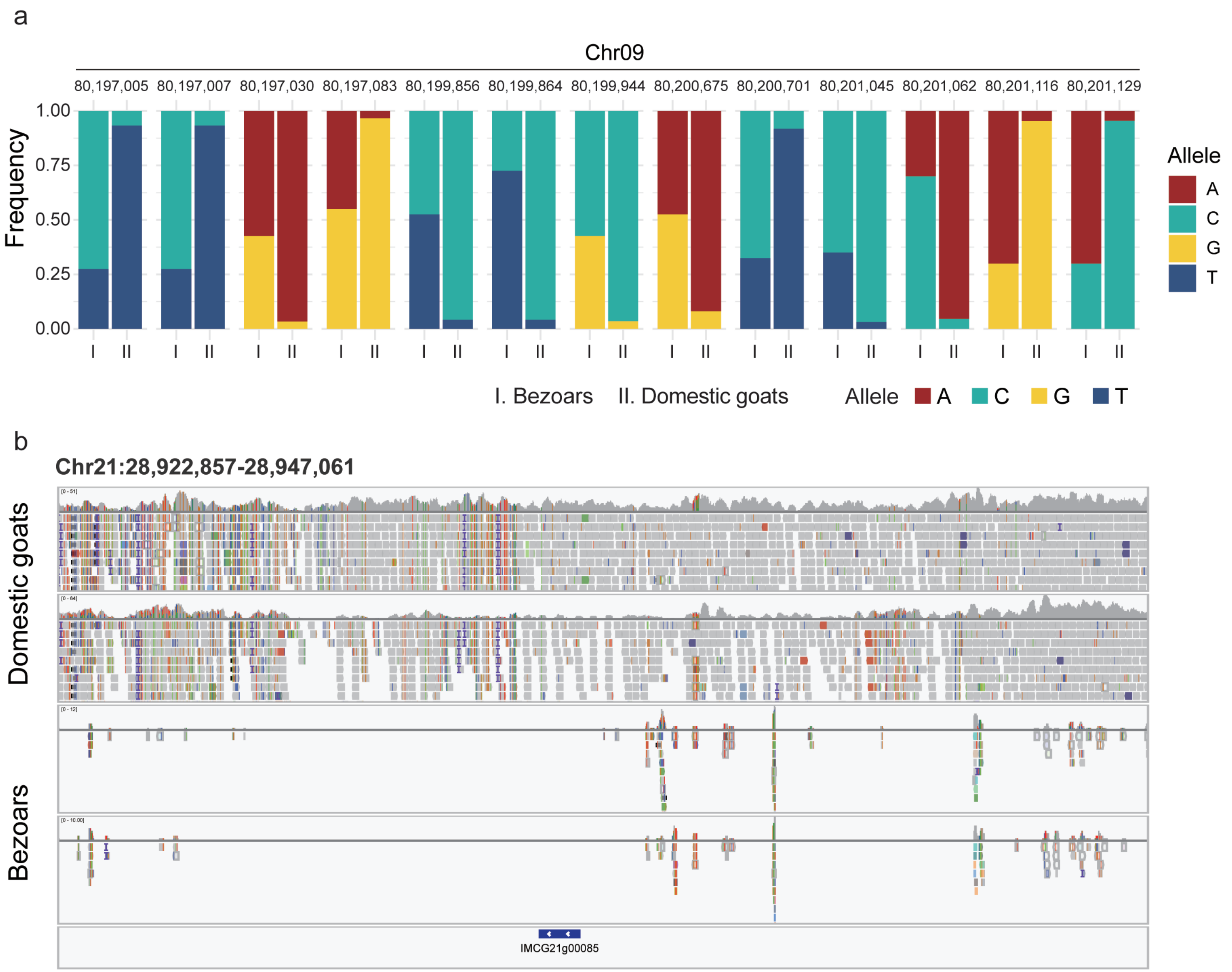

Supplementary Fig. 24 Mutation sites and their frequencies in the domestic and wild (bezoars) goats for *NKG2D* and *MYADM* genes in PURs. a, Thirteen SNP loci in *NKG2D* (IMCG9g00353) under domestication selection showed significant allelic frequency differences between bezoars and domestic goats. **b,** A deletion was found in *MYADM* (IMCG21g00085) of bezoars on the PURs of Chr21, which was under domestication selection.

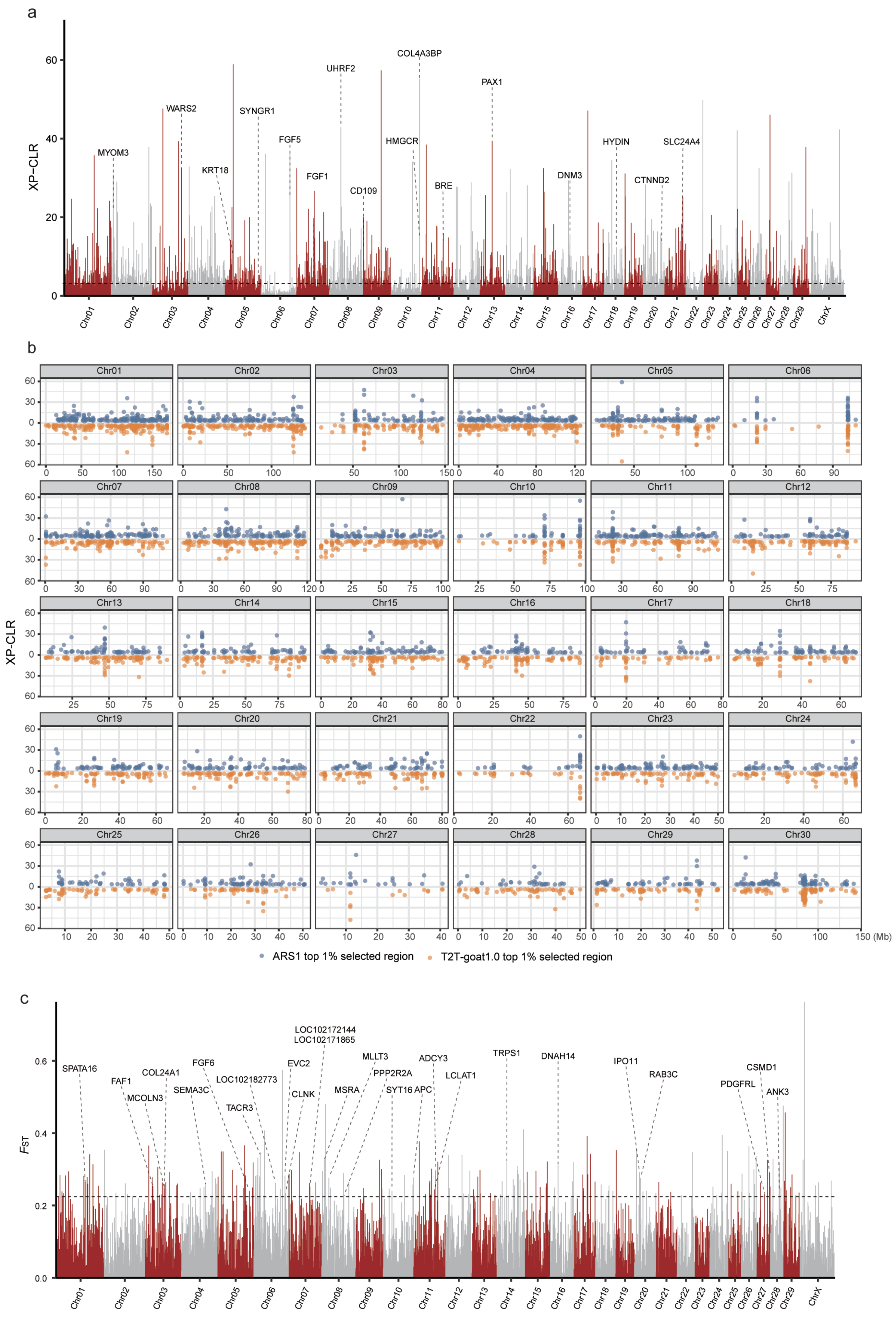

Supplementary Fig. 25 Manhattan plot of selective signals associated with cashmere traits using ARS1 as the reference genome. a, Selective signals between cashmere and non-cashmere goats are determined based on SNPs using top 1% XP-CLR values (horizontal dash line). **b,** Selective signals between cashmere and non-cashmere goats based on top 1% XP-CLR values are compared across the chromosomes between with ARS1 and T2T-goat1.0 as references. **c,** Selective signals between cashmere and non-cashmere goats are determined based on SVs using top 1% F_{ST} values (horizontal dash line). Gene symbols are shown if the genes are identified both by using ARS1 and T2T-goat1.0 as references.

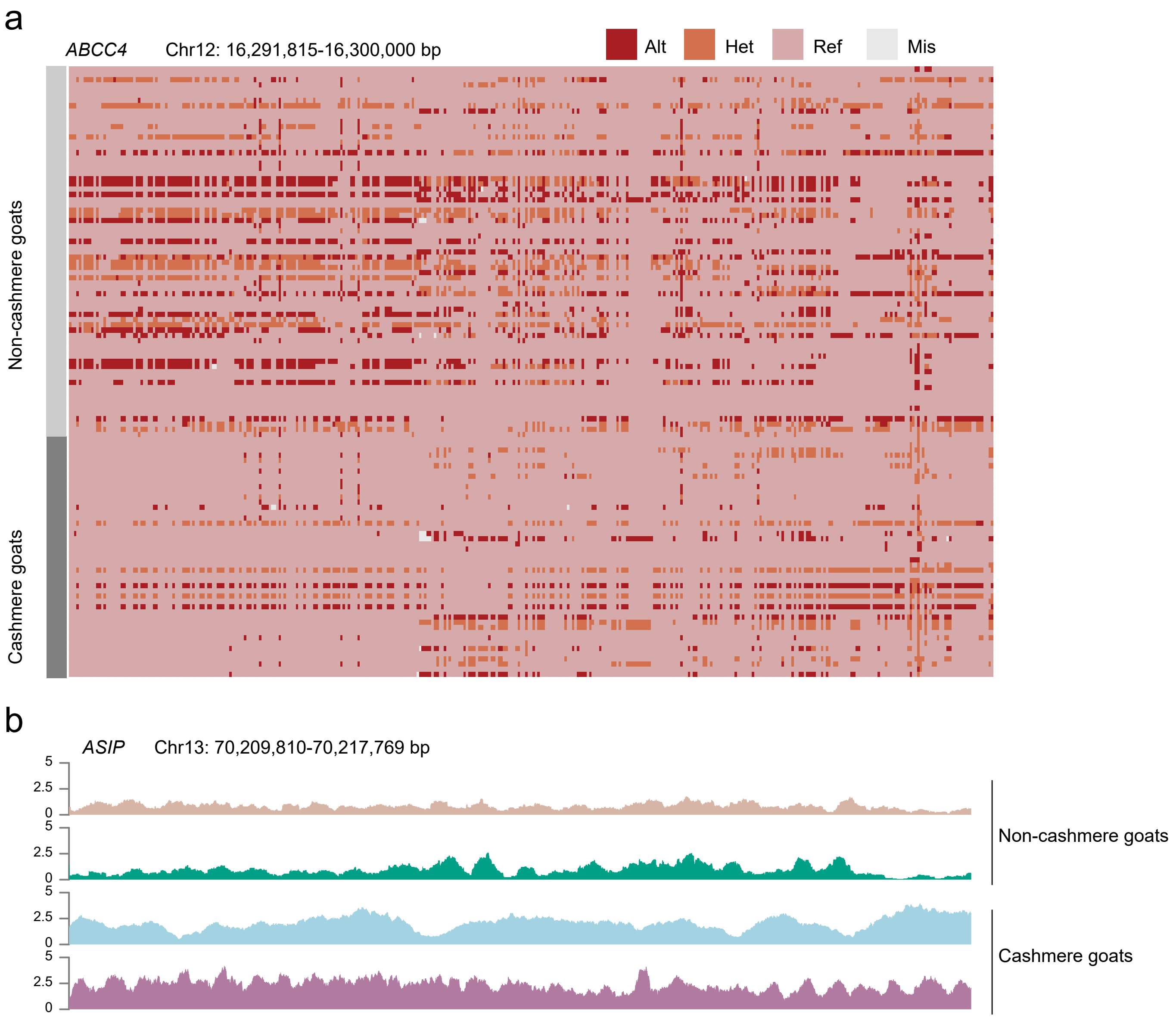

Supplementary Fig. 26 Mutation sites and their frequencies in cashmere and non-cashmere goats for *ABCC4* and *ASIP*. a, SNP loci in *ABCC4* (IMCG12g00092) under the selection of the cashmere trait showing significant allelic frequency differences between cashmere and non-cashmere goats. **b,** A duplication was found in the *ASIP* gene (IMCG13g00511) in cashmere goats, and the coverage of short reads in this region showed significant increase after normalization of total sequenced reads in cashmere goats, compared to non-cashmere goats. The coverage increase suggested more gene copies of *ASIP* gene in cashmere goats.

Supplementary Methods

Sample collection and DNA extraction

The blood from a 4-month buck of Inner Mongolia cashmere goat and their parents was collected from the National Preservation Farm for Arbas Cashmere Goat (latitude 39.208408° N, longitude 107.928814° E, Ordos, Inner Mongolia, China, and used for the T2T genome assembly (Fig. 1a). Additionally, five domestic goats from five representative goat breeds (Liaoning cashmere goat, Zhongwei goat, Jining grey goat, Boer goat, and Tibetan goat) were selected, and their blood was collected for PacBio genome sequencing (Supplementary Table 1). The selected domestic breeds were well known for their specialized traits (Supplementary Figure 16 and Supplementary Table 1), for example, meat, wool, high fertility and skin etc. Liaoning cashmere goat is an elite breed with the highest wool production. Zhongwei goat is mainly used for the production of white fur and leather goods. Jining Grey goat is a highly prolific Chinese native goat breed and typically gives birth twice in one year or three times in two years. Tibetan goat has been well adapted to plateau.

High-molecular-weight (HMW) genomic DNA was extracted from blood based on the CTAB method, and purified with Blood & Cell Culture DNA Kit (Cat# 13343, Qiagen, Beijing, China) following the protocols of manufacture. DNA integrity and concentration were assessed using agarose gels and Qubit 2.0 Fluorometer (Life Sciences, CA). DNA degradation and contamination of the extracted DNA was monitored on 1% agarose gels. DNA purity was then detected using NanoDrop™ One UV-Vis spectrophotometer (Thermo Fisher Scientific, Waltham, MA, USA) and Qubit 3.0 Fluorometer (Invitrogen life Technologies, Carlsbad, CA, USA), of which OD260/280 ranges from 1.8 to 2.0 and OD 260/230 is between 2.0-2.2.

ONT sequencing

The Blue Pippin system (Sage Science, Beverly, MA) was used to retrieve large DNA fragments (> 200 kb) by gel cutting. For the ultra-long Nanopore library, approximately 8–10 μg of genomic DNA was processed using the Ligation Sequencing Kit (Cat# SQK-LSK109, Oxford Nanopore Technologies, Oxford, UK) according the manufacturer's instructions. DNA libraries were constructed and sequenced to generate ultralong ONT reads (N50 > 150 kb) on the PromethION (Oxford Nanopore Technologies) at the Genome Center of Grandomics (Wuhan, China).

PacBio HiFi sequencing

A total amount of 8 μg DNA were sheared by g-TUBEs (Covaris, USA), and purified by using 0.45X AMPure Purification beads (PacBio, Menlo Park, CA). The 20-kb fragments were selected using the BluePippin™ Size Selection System (Sage Science, Beverly, MA, USA), and sequencing libraries were constructed using SMRTbell Express Template Prep Kit 2.0 (Pacific Biosciences, USA). Briefly, DNA fragments were end repaired, A-tailing, and ligated with the hairpin adaptor for PacBio sequencing. The libraries were then treated by nuclease with SMRTbell Enzyme Cleanup Kit and purified by AMPure PB Beads. Target fragments were screened by the PippinHT (Sage Science, USA). The SMRTbell library was then purified by AMPure PB beads, and Agilent 2100 Bioanalyzer (Agilent Technologies, USA) was used to detect the size of library fragments. Sequencing was performed on a PacBio Sequel II instrument with Sequencing Primer V5 and Sequel II Binding Kit 2.2 in the Grandomics (Wuhan, China) by two modes of Continuous Long Reas (CLR) and Circular Consensus Sequencing (CCS; HiFi).

MGI genome sequencing

For MGI genome sequencing, genomic DNA was used to construct the MGI libraries using

MGIEasy Universal DNA Library Prep Kit V1.0 (Cat# 1000005250, MGI, Shenzhen, China) following the standard protocol. Briefly, 1 μg genomic DNA was randomly fragmented by g-TUBEs (Covaris, MA, USA), and the fragmented DNA was selected for an average size of 200-400bp using MGIEasy DNA Clean beads (Cat# 1000005279, MGI). Selected fragments were end-repaired, 3'adenylated and then ligated to adapters. DNA samples were amplified and purified using the MGIEasy DNA Clean beads (Cat# 1000005279, MGI). The double-stranded PCR products were heat denatured and circularized using the splint oligos in MGIEasy Circularization Module (Cat# 1000005260, MGI). The single strand circle DNA (ssCir DNA) were formatted as the final library, and the qualified libraries were sequenced on DNBSEQ-T7RS platform (MGI).

Bionano sequencing

For the Bionano Sequencing, blood samples collected with EDTA was transferred to the lab, and ultra-high molecular weight (uHMW) DNA was extracted using Bionano Prep SP Blood and Cell Culture DNA Isolation Kit (Cat#30033, Bionano Genomics, San Diego, CA, USA). To generate the optical map, uHMW DNA was labeled and stained following the protocol of Bionano Prep Direct Label and Stain (DLS) Kit (Cat#30206, BioNano Genomics). The labeled sample was then loaded onto a Saphyr chip and run on the Saphyr imaging instrument (BioNano Genomics).

Hi-C sequencing

Hi-C (high−throughput chromosome conformation capture technique) libraries were prepared from cross-linked chromatins of white blood cells according to the previous Hi-C protocol[1]. A volume of 0.1 ml blood was cross-linked for 10 minutes with 1% final concentration fresh formaldehyde and quenched with 0.2 M final concentration glycine for 5 minutes. The cross-linked cells were subsequently lysed in lysis buffer, and the extracted nuclei were

resuspended with 150 μL 0.1% sodium dodecyl sulfate (SDS) for incubation at 65°C for 10 minutes. Then SDS molecules were quenched by adding 120 μL water and 30 μL 10% Triton X-100 for a 15-minutes incubation at 37°C. DNA in the nuclei was digested by adding 30 μL 10x New England Biolabs (NEB) buffer and 150 U of *DpnII*, before incubation at 37°C overnight. Next, after *DpnII* enzyme inactivated at 65°C for 20 minutes, the cohesive ends were filled using Klenow at 37°C for 2 hours. Subsequently, proximity ligation was performed using T4 DNA ligase at 16°C for 4 hours, and after ligation, the cross-linking was reversed with 200 μg/mL proteinase K (Thermo Fisher Scientific, Cleveland, OH, USA) at 65°C overnight. DNA was purified using QIAamp DNA Mini Kits (Qiagen), and sheared to an average length of 400 bp. Ligation junctions were pulled down by Dynabeads MyOne Streptavidin C1 (Thermo Fisher Scientific) according to the manufacturer's instructions. The Hi-C library for MGI sequencing was prepared using the MGIEasy Universal DNA Library Prep Kit V1.0 and was sequenced on a MGI-2000 platform (MGI).

Bulk RNA-sequencing and data analysis

Total RNA was extracted from 15 fresh tissues (e.g., testis, liver, lung, and heart, Supplementary Table 1) using TRIzol reagent in RNAprep Pure Tissue Kit (Cat# 4992236, TIANGEN Biotech, Beijing, China). The poly-A RNAs were enriched from total RNA from blood using Dynabeads mRNA Purification Kit (Cat#61006, Invitrogen) and fragmented into small pieces using fragmentation reagent in MGIEasy RNA Library Prep Kit V3.1 (Cat# 1000005276, MGI). The first strand cDNA was synthesized using random primes and reverse transcriptase, followed by second strand cDNA synthesis. The synthesized cDNA was end-repaired, A-tailing added and ligated to the sequencing adapters according to library construction protocol. The cDNA fragments were amplified by PCR and purified with MGIEasy DNA Clean beads (Cat# 1000005279, MGI). Library was analyzed on the Agilent Technologies 2100 bioanalyzer. The double stranded PCR products were heat denatured and

circularized by the splint oligo sequence in MGIEasy Circularization Module (Cat# 1000005260, MGI). The single strand circle DNA (ssCir DNA) were formatted as the final library. The qualified libraries were sequenced on DNBSEQ-T7RS platform. Short-read RNA-Seq libraries for the other 15 tissues were prepared using Illumina Stranded mRNA Prep Kit (Cat# RS-122-2101, Illumina, San Diego, CA, USA), and sequenced on a HiSeq 2000 platform (Illumina) to generate paired-end reads.

Raw RNA-Seq reads were trimmed by using fastp[2] (v0.23.1), with parameters of "-n 0 -f 5 -F 5 -t 5 -T 5". After trimming, the clean data was aligned to the T2T-goat1.0 assembly using STAR[3] (v2.7.9a). FPKM (fragment per kilobase of transcript per million mapped read) value was calculated for each gene using Stringtie[4] (v1.3.4d) with the default parameters.

Genome assembly

The initial genome assembly for the 4-month buck was performed based on 141.03 Gb PacBio HiFi (49.3× coverage) with an N50 of 18.95 kb using Hifiasm[5] (v0.16.1-r375) with default parameters.

```
hifiasm -o genome-assembly -t 40 rsy.hifi.fa.gz
```

Telomere assembly

HiFi reads that contained >10 telomere-specific repeats (i.e., AACCCT or AGGGTT) were pooled together with the ones that were unmapped to the genome assembly, using BLASTN[6] (v2.10.0). The HiFi reads aligned to the 1-Mb chromosomal ends were also pooled with the above HiFi reads to perform telomere assembly using HiFiasm (v0.16.1-r375) with the default parameters. The contigs with telomeric sequences were aligned to GV5 using Minimap2[7] (v2.26), and corrected based on their potential 1-Mb chromosomal ends using the

"correct" command of RagTag (v2.1.0)[8]. Telomeres were placed back at the 1-Mb ends of each chromosome using the scaffold command of RagTag (v2.1.0).

```
#blast searching for three kinds of HiFi reads (telomere repeats in ref.fasta)
makeblastdb -in HiFi.fasta -input_type fasta-dbtype nucl -title CL -out CL_db
blastn -db CL_db -query ref.fasta -out ref-CL.blast
#hifiasm assembly
hifiasm -t 20 -o telomere all.hifi.fa
#ragtag for placing telomeric regions
ragtag.py correct -t 10 --aligner minimap2 ragtag.py goat-chromosome-1Mb-ends.fasta telomere-contigs.fasta
ragtag.py scaffold -t 10 --aligner minimap2 goat-chromosome-1Mb-ends.fasta ./ragtag_output/ragtag.correct.fasta -C
```

Polish pipeline locally developed

The polish pipeline was written based on Cromwell (https://github.com/broadinstitute/cromwell), a Workflow Management System geared towards scientific workflows, which supports the built-in Workflow Description Language (WDL, https://github.com/openwdl/wdl). It employs a total of 18 software or scripts that are third-party, e.g., Nextpolish[9], Nextpolish2[10], yak (https://github.com/lh3/yak), Minimap2, SAMtools[11], etc., or local written scripts in Python language, e.g., bam_filter.py, bam_region_depth.py, and map_one_by_one.py. It involves 8 steps of processing, including (1) *k*-mer generation for the following NextPolish softwares, (2) determination of low-quality regions (LQRs, mostly referring to the gaps) and high-quality regions (HQRs), (3) LQRs mapped for their chromosomal coordinates and orientations, (4) polishing LQRs by NextPolish, (5) polishing LQRs by NextPolish2, (6) polishing high coverage regions (HQRs) by NextPolish2, (7) merging LQRs and HQRs and (8) final polish for the whole genome by NextPolish2. The input data consists of long reads of ONT and PacBio and short reads, and the genome assembly. The configure files (main.wdl, run.json and software.json) should be set up before running the main shell script "run.sh". The output results refer to the polished genome with consensus quality (QV) improved. The whole pipeline is available in GitHub

(https://github.com/Wuhui2024/CAU-T2T-Goat).

```
# Minimap2 alignment
minimap2 –ax –uf –k14 $referenceGenome.fa $sample.fastq | samtools sort -@ 8 -O BAM -o $outfile.bam - && samtools index $outfile.bam
# yak generated 21-mers and 31-mers respectively.
${yak} count -k 21 -t ${cpu} -o ${workdir}/01.sr.short.yak <(zcat ${sep=" " sgs_data[0]})
${yak} count -k 31 -t ${cpu} -o ${workdir}/01.sr.short.yak <(zcat ${sep=" " sgs_data[0]})

# NextPolish2 used 21-mers and 31-mers to conduct polish, based on HiFi alignments using MiniMap2.
${script}/nextPolish2 --out ${workdir}/08.finally_polish.fasta --thread ${cpu} ${workdir}/08.merge.fasta.sort.bam ${mergefasta} ${workdir}/01.sr.long.yak ${workdir}/01.sr.short.yak
```

To evaluate the effects of the polishing above, especially multiple rounds, we improved the QVs from 32.573 for GV5 to 54.1759 for the final assembly T2T-goat1.0, and obtained significantly decreased homozygous SNPs (genotype 1-1) from 234984 to 4974 and single-base error rates from 0.7888% to 0.0174%.

Assembly quality value (QV) calculation

The hash table of *21*-mers was generated for T2T-goat1.0 based on the MGI short reads using the meryl command of Merfin[12] (v1.1), and the consensus base quality value (QV) was calculated using the Merqury[13] (v1.3).

```
# generate 21-mers for pair-end MGI short reads
for i in r1 r2;
do
    meryl k=21 threads=10 memory=50g count output read${i}.meryl M1_0.clean.${i}.fastq.gz
done
meryl  union-sum output goat.k21.meryl  read*.meryl

# running merqury
merqury-1.3/merqury.sh  goat.k21.meryl  goat.genome.fasta  goat
```

Minimum unique *k*-mer (MUK) calculation

MUKs were calculated in 100-kb windows for both T2T-goat1.0 and ARS1, according to the previously published method[14]. T2T Minimum Unique *K*-mer Analysis pipeline (https://github.com/msauria/T2T_MUK_Analysis) was used with a minor modification to identify the longest common prefix (LCP) based on the sequences concatenated from the two genome assemblies. LCP values plus one represented the MUK at a window site (100-kb windows), and the windows with gaps in *ARS1* were determined for no MUK.

Previously unresolved regions (PURs)

The chromosomes and all sequences of ARS1 genome assembly (GenBank accession no. GCF_001704415.1) were aligned to T2T-goat1.0 using Winnomap2[15] (v2.03), and the PURs and newly assembled regions (NARs) were summarized using BEDTools[16] (v2.30.0) respectively.

```
# 01 winnomap2 alignment
winnowmap -ax asm20 -t 20 -H --MD goat-genome.fasta ARS1.genome.fasta/
ARS1.chromosomes.fasta > out.nocontig.sam
# 02 bam converted into paf
paftools.js sam2paf -p out.nocontig.sam > out.paf
# 03 get PURs or NARs
cat out.paf |awk '{if ($12 > 0) print $6"\t"$8"\t"$9}' |bedtools sort -i -
|bedtools merge -i - |bedtools complement -i - -g goat-genome.chr.len >
out.pur.region
```

Segmental duplications (SDs) and other repeats in PURs

SDs and other repeats in PURs were discovered by overlapping the bed files and PURs using BEDTools (v2.30.0).

```
# merging all files of SDs
bedtools merge -i all.sd.bed > sd.bed
# overlapping SDs and PURs
bedtools intersect -a pur.bed -b sd.bed


#01 satelite_cen
#sort the centromeric satellite regions (final.Sat_centr.region)
```

```
sort -k1,1 -k2,2n unsorted.Sat_centr.region >Sat_centr.region
#sort PURs (pur.region)
sort -k1,1 -k2,2n unsorted.pur.region > pur.bed
# overlapping SDs and PURs for the file (sac_pur.bed)
bedtools intersect -a pur.bed -b Sat_centr.region >sac_pur.bed
# making bed file (nosac_pur.bed) for PURs without SDs
bedtools subtract -a pur.bed -b Sat_centr.region > nosac_pur.bed


#02 SDs in PURs
# merging all files of SDs
bedtools merge -i all.sd.bed > sd.bed
# overlapping SDs and PURs
bedtools intersect -a pur.bed -b sd.bed > sd_pur.bed
# bed regions with the overlapped SDs and centromeric satellite regions
bedtools intersect -a sac_pur.bed -b sd_pur.bed >sd_sac_pur.bed

# only centromeric satellite regions without SDs
bedtools subtract -a sac_pur.bed -b sd_sac_pur.bed > sac_nosd.bed
# only SDs without centromeric satellite regions
bedtools subtract -a sd_pur.bed -b sd_sac_pur.bed > sd_nosac.bed


#03 rDNA in PURs
# retrieving PURs without centromeric satellite regions and SDs
bedtools subtract -a nosac_pur.bed -b sd_pur.bed >nosac_sd_pur.bed
# retrieving rDNAs in PURs without centromeric satellite regions and SDs
bedtools intersect -a nosac_sd_pur.bed -b rDNA.bed > rDNA_nosa_sd.bed


#04 Repeats by RepeatMasker
# merging all repeats by RepeatMasker
bedtools merge -i sort.repeat.bed >repeat.bed
# retrieving the PURs without centromeric satellites, SDs, and rDNA.
bedtools subtract -a nosac_sd_pur.bed -b rDNA.bed >nosac_sd_rdna.pur.bed
# only repeats by RepeatMasker in PURs
bedtools intersect -a nosac_sd_rdna.pur.bed -b repeat.bed >repeat_pur.bed


#05 others
bedtools subtract -a pur.bed -b Sat_centr.region >tmp1
bedtools subtract -a tmp1 -b sd_pur.bed >tmp2
bedtools subtract -a tmp2 -b rDNA.bed >tmp3
bedtools subtract -a tmp3 -b repeat.bed >final_others_pur.bed
```

Gene family and comparative analysis

Orthogroups of protein-coding genes were found in T2T-goat1.0 using OrthoFinder[17] (v2.5.5) with default parameters. The genes' copies were compared among the reference assemblies of *T2T-goat1.0*, *X*inong Saanen Dairy goat ASM2665220v1 (GenBank accession no.

GCA_026652205.1) and ARS1.

Centromere-specific satellite identification

The candidate centromere regions were retrieved based on the blastn results of known 816-bp centromeric satellite DNA (GenBank accession no. U25964.1). A *k*-mer library was generated based on the above deduced centromere regions, using KMC[18] (v3.1.1).

```
kmc -fm -k151 -t16 -ci10 -cs1000000 centromere.fasta count.kmc tmp_dir
kmc_dump count.kmc count.txt
```

Based on the centromeric *k*-mers and their frequencies, 21 kinds of centromeric repeat units were identified using the program SRF (spectral repeat finders)[19].

```
srf -p prefix count.txt > srf.fa
```

All the satellite repeats were blasted against themselves to group them using BLASTN[6] (v2.10.0).

```
Blastn -query srf.fa -subject srf.fa -out srf-srf.blast -word_size 15
```

The three satellite unites were identified based on the three groups of sequence similarities.

SatII: prefix#circ1-702
SatI:

```
prefix#circ6-3237
prefix#circ9-4067
prefix#circ20-3262
prefix#circ11-4080
prefix#circ15-3235
prefix#circ17-3233
prefix#circ16-1632
prefix#circ14-815
prefix#circ10-816
```

SatIII:

```
prefix#circ21-22
prefix#circ19-352
prefix#circ18-418
prefix#circ13-44
prefix#circ7-1760
prefix#circ2-132
```

```
prefix#circ5-154
prefix#circ4-132
prefix#circ12-44
```

The goat Y-chromosome centromere (CenY) (1473 bp) showed very high sequence similarity to the sheep CenY (2516 bp). The four satellite repeat units (SatI, SatII, SatIII and CenY) were aligned to T2T-goat1.0 to assess the satellite contents using BLASTN[6] (v2.10.0).

```
Blastn -query sat_units.fa -db goat-genome -out sat.goatgenome.blast
```

The three newly identified satellite repeats are shown below.

SatI sequence

>Goat_SatI
CGAGAGCACCTGCCGCAACTCGAGAAAATCCAGGAGGTTCTCCCCTCCAGGCGACATGAGGC
CCATTTCCGCTGAGGTATCTCGAGGCTAATCACACCTTACCTCTGGAACTTCCAAAGGGTCCTT
CACACCCTTGCTGCAACTCAAGAAGTACCCCGACATACCCGTCTCCACTCGAGAGGAAGCAG
GAAAGTGCCGCCCACATCAAGAGGAGCCACGTTTCCGCCTCCTAGCTCGAGAGGTTTGATCCT
TTCCCTGCGTGGTGGGGAAAGAATTCGCGGCGTTCCCGTCGCATCTCAAGAGGAGGCGCTCTC
CAGAGGAAAAGCGAGAGGAACTCCAGGTTCGTGCCACCATTGCCAGAGTCCCCCAGATGTCT
CAGTCCATTCCAGGGAACCTGGTTTCCCTGCACTGCCTCGACTTTTAAGCCGAGGATCGACTC
ACACCACTGTGGCACGTGGGAAAGCACTGTGGGAAAGCCTCGTGGGAAAGCCTCATGGGAAA
GTCTAGAGGGAAAGCCACAGATCCCTTGATCCACGAAAAGGGAAGCGTGACACTGCTGCTAC
AGCTCGGGAGGAAAGCGCACGTGCATGCCCCCACTCGAGACGAGGACTTACGCCTCTGGGGA
GACTACAGAAGTCCCCGAAGATCCATGTCGGCACTGGAGAGGAATCCTCAGGTTCCGGCACTG
ACTCCACACAAGGTCTTAGGCCCCGGCATCGACGGGAGAGGAATCCCGAGAGGCCCCCGAGC
AACTCGCATGGGGACTGGGCTTTCCTGAGGCCACCAGAGTGGGTCCCTGAGGTCCCCGTCGTA
AGT

SatII sequence

>Goat_SatII
CTGATCCTGCGTGTCTGGGTTCCTGCCCACAGCCCCAACTCAGGGGAAGCAGCCCCTGAGGCA
AGACCTGCTGGAGGAGCCCTGCGCCCCCGCCTGATAGGCGAGTGCACAGCCAGCAGGGAAGC
GAGCCTCCTCGGGGGAAGAGGAGGCAGCAAGTCCCCGTCAAGCTGAAATCCCTGCTTACACG
AAGCTAAGTGCGACTCCAGTACGGCAGATCTCTAGCTCTGTTCAGAGGCCCGATCTGGCAGAG
TTCCTGGCACCAAGATGCTCTGCCATAGGCGAGCCTTGCGCAGACTTGCCTTGCTTTAAGGCTC
CCTCCCCAGGAAGGAGCAGGCTGTGTGAGGCTCGGAAACGCCACTGGCGCCTTCCTGGCTTC
AGCTCTACCCCTGCCTTGCCTTTGCACTTAGGTCCCAAGCGTCCTTGAGGAGGGCTTAGGGGC
TGGTGGGTTGCACACTGCCAAGGGCTCCAGCAGTGAAAGCTCTGTCAGAGGGAAGCGAAGGG
CAACAGCAGGGCTCCTTCAGCGGTTCCTTGGCCGCCCAGACAAAGCTCCGCACGGAGAGCAC
CAGGTTCCAAGGGCCCCTGGCTGGGTTTTGCATCCCCACACTCCCAGAGGCTGACTCCGTGGG
TGCCTAGAAAGTGTGTGAGAGACGTAGGTTCCCTAGCACTCTCCTCCCCAGCAGAGACATGTG
CAGGTGGTTGGGGC

SatIII sequence

>Goat_SatIII-1
ATCACGTGGGCCCCCAGGGGCA
>Goat_SatIII-2
ATCACGTGGGCCCCCAGGGGAA

CenY sequence

```
>Goat_CenY
GCGCCGCGCCACTCGCGCAGGATGTGGGTGCACGCGCCGCAGGTGGCTGGGGGGCCCCGCGT
GCGCAAGAAGAGAGACGTCGCTCCCTGCAGCGTGGGCAGACGGGTGTCGCGCCTCGCCTGCA
GGTGGACACTGCGCCCACCGGAATGGGGGTTCAGAGACAATGGGGGCACGCGCCTCTCGCGC
CAGAAGAAGGACTGGTTCCCCGGCCGCGCGCCTTGGGGTCTCTTCCGCCTTAGGGTCAGGGC
ACTGGAAGCGCTCCGGCTGCTGCGAGCGGTCTGGAGGGTGGGAGGGGCGGGGAGAGGCGCG
CTTGGGACCTCCGCCGCCCGTGGGGAGTAGGAAGTTGCGGGCTATGGCTGCAGCTGGCGGTG
ACCTGGAGGGGCGCGGGGGTGTGTGGCGCTCCCCGTGGGGCCAGAGGTCCGGCTGGGGCGGC
GGCTCGAGGCGCCCGCGCCCCCCGGGCGACAGGCTCGGCAGGTGAGGAGCCAGAGCGCTCCT
GTTGGCACGCGGGGCCGGCCGGCGCCCCAGAGGTTGTTCTCGCGGAACCCTGGAGACCCGCG
ACCACGTAGGTGAGGCCGGAAGGGCCCAGAAGTCCCCGCAGCGTTCCTGGTCCCGCCCGCCA
GTCCCCTGTGCTCCCCGCAGCGGGCTGGGGATGCCGGACCCCTGAACCCTCTGAGCGCGCCCC
CGCGGCGGGGCCAAAGAGCTGGCCGTCAGGCGGTGTTCGGAAGTCGGGAGGCAGCAGAAGG
GGTCTTGCTCTCCCCCGGGGGCTGCCGGTACACCCTCAGACTGCCCAGTGCGGCGCGCGGCCC
CAATGGCGAATGGCCTCCGTTGGCGCTGCCTGTAGTGTGCGCTCAGACCCTGCGGGCGCTTTG
GCACAAGTGTGCGACGACGATGGCAGCTGGTATATGACTGTTCTATAGCTGGGCGCTCGTGAAT
ACCTGATGGCGATAAGAAGCCTTAGCCCCAAGTGTTGCGCAGGAAGAAGGTGGCAAGGTCAA
GCCGGGAGAGCACAGACAGAACGGTGAGTCAGAGGTCCTGACAGGTCCCAGAGGAGGTCGC
GCCAGGACCTGCATCTCAGCACGAGTGCCAGCGTCTTTAGAGATGAGGAGCGGGATTTGTATT
CTGCGGTTCTGTGGCGTCTTACCCAGACACCTGGGGAAAGCGCGCCAGGACAGAAGGACAGC
GGTGACTTCCCCTTTAAGGACTCCAAGCTCATACTGATAGCCGAAAGTCCCCCCCCCCCCCCC
CCCCGCCTCCCTACCGCCACCGCCCCCCCCCCCGCGCCCCCCCGGGTATTAGCATGAAAGACTT
CTCCTTTGTAAAAGGAGTCTCCAGGAGGGCGCCTGTGAGAAGCAGGCTCTGGTGGACTTGGG
CTGCGGCGGGTCCACTCAAGCGCACACAACTCAGTAGAGGCACGTGCTGGATGGTATCAGTGT
TGACATCCTGCTTGTGGTAGTGTGCTCTGTGCTTCCGCTC
```

Phylogenetic tree analysis of *TSPY* genes

All the protein-coding *TSPY* genes were used to build the phylogenetic tree, with human two *TSPY* genes[20] (ENSP05220103496 and ENSP05220102576) as the outgroup. The multiple protein sequence alignment was performed using MAFFT[21]. The maximum-likelihood tree was built under a HKY nucleotide substitution mode using IQ-TREE[22] (v2.2.2.6), and plotted in iTOL[23] (v6.8.1).

Digital Droplet PCR (ddPCR)

MC1R and *SRY* were selected as reference genes to estimate the copy number of *TSPY* and *HSFY*, respectively. Primers and probes for ampliconic genes were designed (Supplementary Table 10) using Primer Premier 5.0. Briefly, DNA was digested with HindIII enzyme by incubating at 37°C for 60 minutes, followed by enzyme inactivation at 80°C for 20 minutes, resulting in digested products. For *HSFY* copy number assay, the 20 µl digital PCR reaction mixture consisted of 10 µl ddPCR Supermix, 7.2 µl primers, 1 µl probes, 1 µl of the digested

product, and 0.8 µl ddH_2O. Master mixes were then emulsified with 70 µl droplet generator oil using a droplet generator according to the manufacturer's instructions. After droplet generation, the reaction plate was placed in a Bio-Rad PCR machine for amplification with the following program: 10 minutes at 95°C for initial denaturation, followed by 40 cycles of 30 seconds at 94°C for denaturation and 1 minute at 60°C for extension, 10 minutes at 98°C, and held at 4°C. For *TSPY* copy number, each reaction consists of 10 µl Q200 ddPCR EvaGreen Supermix, 0.4 µl primers, 1 µl DNA, and 8.6 µl ddH_2O. After droplet generation, the reaction plate was placed in a Bio-Rad PCR machine for amplification with the following program: 5 minutes at 95°C for initial denaturation, followed by 40 cycles of 30 seconds at 95°C, 1 minute at 60°C and 15 seconds at 72°C, and held at 4°C.

References

1. Li, N. et al. Super-pangenome analyses highlight genomic diversity and structural variation across wild and cultivated tomato species. *Nat. Genet.* **55**, 852-860 (2023).
2. Chen, S., Zhou, Y., Chen, Y. & Gu, J. fastp: an ultra-fast all-in-one FASTQ preprocessor. *Bioinformatics* **34**, i884-i890 (2018).
3. Dobin, A. et al. STAR: ultrafast universal RNA-seq aligner. *Bioinformatics* **29**, 15-21 (2013).
4. Shumate, A., Wong, B., Pertea, G. & Pertea, M. Improved transcriptome assembly using a hybrid of long and short reads with StringTie. *PLoS Comput. Biol.* **18**, e1009730 (2022).
5. Chen, J. et al. A complete telomere-to-telomere assembly of the maize genome. *Nat. Genet.* **55**, 1221-1231 (2023).
6. Camacho, C. et al. BLAST+: architecture and applications. *BMC Bioinformatics* **10**, 421 (2009).
7. Li, H. Minimap2: pairwise alignment for nucleotide sequences. *Bioinformatics* **34**, 3094-3100 (2018).
8. Alonge, M. et al. Automated assembly scaffolding using RagTag elevates a new tomato system for high-throughput genome editing. *Genome Biol.* **23**, 258 (2022).
9. Hu, J., Fan, J., Sun, Z. & Liu, S. NextPolish: a fast and efficient genome polishing tool for long-read assembly. *Bioinformatics* **36**, 2253-2255 (2020).
10. Hu, J. et al. NextPolish2: a repeat-aware polishing tool for genomes assembled using HiFi long reads. *bioRxiv*, 2023.04. 26.538352 (2023).
11. Danecek, P. et al. Twelve years of SAMtools and BCFtools. *Gigascience* **10**, giab008 (2021).
12. Formenti, G. et al. Merfin: improved variant filtering, assembly evaluation and polishing via *k*-mer validation. *Nat. Meth.* **19**, 696-704 (2022).
13. Rhie, A., Walenz, B.P., Koren, S. & Phillippy, A.M. Merqury: reference-free quality, completeness, and phasing assessment for genome assemblies. *Genome Biol.* **21**, 245

(2020).
14. Aganezov, S. et al. A complete reference genome improves analysis of human genetic variation. *Science* **376**, eabl3533 (2022).
15. Jain, C., Rhie, A., Hansen, N.F., Koren, S. & Phillippy, A.M. Long-read mapping to repetitive reference sequences using Winnowmap2. *Nat. Meth.* **19**, 705-710 (2022).
16. Quinlan, A.R. & Hall, I.M. BEDTools: a flexible suite of utilities for comparing genomic features. *Bioinformatics* **26**, 841-842 (2010).
17. Emms, D.M. & Kelly, S. OrthoFinder: phylogenetic orthology inference for comparative genomics. *Genome Biol.* **20**, 238 (2019).
18. Kokot, M., Długosz, M. & Deorowicz, S. KMC 3: counting and manipulating *k*-mer statistics. *Bioinformatics* **33**, 2759-2761 (2017).
19. Zhang, Y., Chu, J., Cheng, H. & Li, H. *De novo* reconstruction of satellite repeat units from sequence data. *Genome Res.* (2023).
20. Rhie, A. et al. The complete sequence of a human Y chromosome. *Nature* **621**, 344-360 (2023).
21. Katoh, K. & Standley, D.M. MAFFT multiple sequence alignment software version 7: improvements in performance and usability. *Mol. Biol. Evol.* **30**, 772-780 (2013).
22. Minh, B.Q. et al. IQ-TREE 2: new models and efficient methods for phylogenetic inference in the genomic era. *Mol. Biol. Evol.* **37**, 1530-1534 (2020).
23. Letunic, I. & Bork, P. Interactive Tree Of Life (iTOL) v5: an online tool for phylogenetic tree display and annotation. *Nucleic Acids Res.* **49**, W293-W296 (2021).
