# Supplementary material for: Telomere-to-telomere genome assembly of a male goat reveals variants associated with cashmere traits
Source: Nat Commun. 2024 Nov 20;15:10041. doi: 10.1038/s41467-024-54188-z (PMC11579321; doi:10.1038/s41467-024-54188-z)
Supplement: Supplementary file 2 — Reporting Summary [file 41467_2024_54188_MOESM2_ESM.pdf]

nature portfolio

Corresponding author(s): Menghua Li
Last updated by author(s): Sep 30, 2024

Reporting Summary

Nature Portfolio wishes to improve the reproducibility of the work that we publish. This form provides structure for consistency and transparency in reporting. For further information on Nature Portfolio policies, see our Editorial Policies and the Editorial Policy Checklist.

Statistics

For all statistical analyses, confirm that the following items are present in the figure legend, table legend, main text, or Methods section.

n/a	Confirmed	
[ ]	[x]	The exact sample size (*n*) for each experimental group/condition, given as a discrete number and unit of measurement
[ ]	[x]	A statement on whether measurements were taken from distinct samples or whether the same sample was measured repeatedly
[ ]	[x]	The statistical test(s) used AND whether they are one- or two-sided<br>*Only common tests should be described solely by name; describe more complex techniques in the Methods section.*
[x]	[ ]	A description of all covariates tested
[x]	[ ]	A description of any assumptions or corrections, such as tests of normality and adjustment for multiple comparisons
[ ]	[x]	A full description of the statistical parameters including central tendency (e.g. means) or other basic estimates (e.g. regression coefficient) AND variation (e.g. standard deviation) or associated estimates of uncertainty (e.g. confidence intervals)
[ ]	[x]	For null hypothesis testing, the test statistic (e.g. *F*, *t*, *r*) with confidence intervals, effect sizes, degrees of freedom and *P* value noted<br>*Give P values as exact values whenever suitable.*
[x]	[ ]	For Bayesian analysis, information on the choice of priors and Markov chain Monte Carlo settings
[x]	[ ]	For hierarchical and complex designs, identification of the appropriate level for tests and full reporting of outcomes
[x]	[ ]	Estimates of effect sizes (e.g. Cohen's *d*, Pearson's *r*), indicating how they were calculated

Our web collection on statistics for biologists contains articles on many of the points above.

Software and code

Policy information about availability of computer code

Data collection	PacBio HiFi data were generated by PacBio Sequel II platform. Ultralong ONT data were generated by Nanopore PromethION platform. NGS data were generated by MGI DNBSEQ-T7RS platform. Iso-seq data were generated by PacBio Sequel II platform. RNA-seq data were generated by MGI DNBSEQ-T7RS platform and Illumina HiSeq 2000 platform. ChIP-seq data were generated by Illumina NovaSeq-6000 platform. Hi-C data were generated by MGI DNBSEQ-T7RS platform. Bionano data were generated by Bionano Genomics Saphyr System.<br>The whole-genome sequence data of caprine genomes, which included 448 domesticated and 68 wild goat individuals, were downloaded from public databases.
Data analysis	All custom scripts used in this study will be publicly available at https://github.com/Wuhui2024/CAU-T2T-Goat.<br>The softwares that were used in this manuscript are shown as followed:<br>(1) Initial assembly: Hifisam v0.16.1-r375, BLASTN v2.10.0<br>(2) Bionano scaffolding: Bionano Solve software v3.5.1<br>(3) Pseudochromosome construction: HiC-Pro v2.8.1, Bowtie2 v2.3.2, LACHESIS<br>(4) Gap verification and filling: IGV v2.13, Minimap2 v2.23, NextDenovo v2.5.2<br>(5) Initial assembly of the Y chromosome: Jellyfish v2.3.0, Minimap2 v2.23, NextDenovo v2.5.2, Hifisam v0.16.1, MUMmer v4.0.0<br>(6) Telomere filling: BLASTN v2.10.0, Minimap2 v2.26, SAMtools v1.18, Hifiasm v0.16.1, RagTag v2.1.0<br>(7) Genome polishing: Minimap2 v2.26, NextPolish2 v0.1.0, NextPolish v1.4.1<br>(8) Genome assessment and validation: BWA v0.7.17, karyoploteR v1.8.4, Merqury v1.3.1, BUSCO v4.0.5, SAMtools v1.18, BCFtools v1.15.1<br>(9) Repeat annotation: GMATA v2.2, Tandem Repeats Finder v4.09.1, MITE-Hunter v1.0, RepeatModeler2 v2.0.4, RepeatMasker v4.1.4<br>(10) Segmental duplication (SD) identification: BISER v1.4, Circos v0.69<br>(11) Protein-coding gene annotation: STAR v2.7.9a, Stringtie v1.3.4d, Minimap2 v2.16, PASA v2.5.2, GeMoMa, AUGUSTUS v3.3.1,

EvidenceModeler v1.1.1, TransposonPSI v1.0.0, MCScanX v1.0.0, IGV-GSAman v0.6.84
(12) Methylation by PacBio and ONT long reads: pbmm2 v1.13.0, pb-CpG-tools v2.3.1, GuPPy v6.1.2, Nanopolish v0.14.0, BEDTools v2.31.0, karyoploteR v1.8.4
(13) Identification and validation of centromeric regions: fastp v0.23.1, Bowtie2 v2.4.2, MACS3 v3.0.0b2, BEDTools v2.30.0, karyoploteR v1.8.4, StainedGlass v0.5, SRF, NeSSie, StainedGlass (v0.5)
(14) Repeat identification within centromeres: BEDTools v2.30.0, BLASTN v2.10.0
(15) SV identification based on long-read sequences: Sniffles v2.0.6, cuteSV v2.0.1, pbsv v2.9.0, Minimap2 v2.26, SAMtools v1.18, Mosdepth v0.3.4, SURVIVOR v1.0.7
(16) SNP and SV calling based on short-read sequences: Trimmomatic v0.39, BWA v0.7.17-r1188, SAMtools v1.16, GATK v4.3, VCFtools v0.1.16, SnpEff v5.1d, Delly v0.8.7, Manta v1.6.0, Smoove v0.2.8, SURVIVOR v1.0.7
(17) Genetic diversity and population structure: VCFtools v0.1.16, PLINK v2.00a3.7, EIGENSOFT v8.0.0, ADMIXTURE v1.3.0, VCF2Dis v1.47, TreeBeST v1.9.2, iTOL v6.8.1, SplitsTree v6.3.27
(18) Selective sweeps: XP-CLR v1.1.2, VCFtools v0.1.16, ggplot2 v2.1.1

For manuscripts utilizing custom algorithms or software that are central to the research but not yet described in published literature, software must be made available to editors and reviewers. We strongly encourage code deposition in a community repository (e.g. GitHub). See the Nature Portfolio guidelines for submitting code & software for further information.

Data

Policy information about availability of data

All manuscripts must include a data availability statement. This statement should provide the following information, where applicable:

- Accession codes, unique identifiers, or web links for publicly available datasets
- A description of any restrictions on data availability
- For clinical datasets or third party data, please ensure that the statement adheres to our policy

The genome assemblies T2T-goat1.0, T2T-goat1.0P, and T2T-goat1.0M; and T2T-goat2.0, T2T-goat2.0P, and T2T-goat2.0M are available in NCBI under accession numbers GCA_040806595.1, GCA_041920685.1, GCA_041736475.1, GCA_041735815.1, GCA_041053135.1, and GCA_040970035.1, respectively. Raw sequencing data generated in this study, including PacBio HiFi data, ultralong ONT data, MGI data, Iso-seq data and ChIP-seq data, can be achieved from National Genomics Data Center (https://ngdc.cncb.ac.cn/) under BioProject number PRJCA022847, and NCBI under BioProject number PRJNA1062519. The details of data mentioned above and other publicly available data downloaded in this study are provided in Supplementary Data 1, 5 and 19. T2T-goat2.0 is the update of T2T-goat1.0, with all the centromeres placed on the leftmost ends of acrocentric autosomes. Source data are provided with this paper.

Research involving human participants, their data, or biological material

Policy information about studies with human participants or human data. See also policy information about sex, gender (identity/presentation), and sexual orientation and race, ethnicity and racism.

Reporting on sex and gender	No human samples are included in this study.
Reporting on race, ethnicity, or other socially relevant groupings	No human samples are included in this study.
Population characteristics	No human samples are included in this study.
Recruitment	No human samples are included in this study.
Ethics oversight	No human samples are included in this study.

Note that full information on the approval of the study protocol must also be provided in the manuscript.

Field-specific reporting

Please select the one below that is the best fit for your research. If you are not sure, read the appropriate sections before making your selection.

[x] Life sciences [ ] Behavioural & social sciences [ ] Ecological, evolutionary & environmental sciences

For a reference copy of the document with all sections, see nature.com/documents/nr-reporting-summary-flat.pdf

Life sciences study design

All studies must disclose on these points even when the disclosure is negative.

Sample size	A 4-month-old buck of the Inner Mongolia cashmere goat was used for T2T genome assembly, together with their parents for assistance of chromosome Y assembly. 14 tissues from three Inner Mongolia cashmere goats were sampled. Five goats, including Liaoning cashmere goat, Zhongwei goat, Jining gray goat, Boer goat, and Tibetan goat were sampled for long-read SV calling. A total of 516 individuals were used for population analyses.
Data exclusions	No data were excluded from the analyses.
Replication	Replications for each analyses were clearly stated in Supplementary materials and Methods.

Randomization	The sampling process for DNA sequencing, RNA-seq, Iso-seq and ChIP-seq was randomly conducted.
Blinding	Blinding is not necessary for T2T genome sequencing and assembly. No blinding should not affect interpretation as all our experiment measures were objective.

Reporting for specific materials, systems and methods

We require information from authors about some types of materials, experimental systems and methods used in many studies. Here, indicate whether each material, system or method listed is relevant to your study. If you are not sure if a list item applies to your research, read the appropriate section before selecting a response.

Materials & experimental systems

n/a	Involved in the study	
☐	☒	Antibodies
☒	☐	Eukaryotic cell lines
☒	☐	Palaeontology and archaeology
☐	☒	Animals and other organisms
☒	☐	Clinical data
☒	☐	Dual use research of concern
☒	☐	Plants

Methods

n/a	Involved in the study	
☐	☒	ChIP-seq
☒	☐	Flow cytometry
☒	☐	MRI-based neuroimaging

Antibodies

Antibodies used	Phospho-CENP-A (Ser7) rabbit polyclonal antibody (Cat# 2187, Cell Signaling Technology, Beverly, MA, USA)
Validation	Details about the validation of the antibody, relevant citations and antibody profiles can be found at https://www.cellsignal.com/products/primary-antibodies/phospho-cenp-a-ser7-antibody/2187.

Animals and other research organisms

Policy information about studies involving animals; ARRIVE guidelines recommended for reporting animal research, and Sex and Gender in Research

Laboratory animals	we collected blood of a 4-month-old buck of the Inner Mongolia cashmere goat and his parents for T2T genome sequencing and assembly. 14 tissues from three Inner Mongolia cashmere goats were sampled. We also collected blood samples from a Liaoning cashmere goat, a Zhongwei goat, a Jining gray goat, a Boer goat, and a Tibetan goat for PacBio sequencing.
Wild animals	There are no wild animals in this study.
Reporting on sex	Inner Mongolia cashmere goat: Male<br>Liaoning cashmere goat: Female<br>Zhongwei goat: Female<br>Jining gray goat: Female<br>Boer goat: Female<br>Tibetan goat: Female
Field-collected samples	There are no field-collected samples in this study.
Ethics oversight	All experimental protocols in this study were reviewed and approved by the Institutional Animal Care and Use Committee of China Agricultural University (CAU20160628-2).

Note that full information on the approval of the study protocol must also be provided in the manuscript.

Plants

Seed stocks	*Report on the source of all seed stocks or other plant material used. If applicable, state the seed stock centre and catalogue number. If plant specimens were collected from the field, describe the collection location, date and sampling procedures.*
Novel plant genotypes	*Describe the methods by which all novel plant genotypes were produced. This includes those generated by transgenic approaches, gene editing, chemical/radiation-based mutagenesis and hybridization. For transgenic lines, describe the transformation method, the number of independent lines analyzed and the generation upon which experiments were performed. For gene-edited lines, describe the editor used, the endogenous sequence targeted for editing, the targeting guide RNA sequence (if applicable) and how the editor was applied.*
Authentication	*Describe any authentication procedures for each seed stock used or novel genotype generated. Describe any experiments used to assess the effect of a mutation and, where applicable, how potential secondary effects (e.g. second site T-DNA insertions, mosiacism, off-target gene editing) were examined.*

ChIP-seq

Data deposition

☒ Confirm that both raw and final processed data have been deposited in a public database such as GEO.

☒ Confirm that you have deposited or provided access to graph files (e.g. BED files) for the called peaks.

Data access links<br>*May remain private before publication.*	Raw data generated by ChIP-seq can be found at the Genome Sequence Archive in National Genomics Data Center (https://ngdc.cncb.ac.cn/) under the BioProject accession number PRJCA022847 and NCBI under the BioProject accession number PRJNA1062519.<br>File for the called peaks are available at GitHub (https://github.com/Wuhui2024/CAU-T2T-Goat).
Files in database submission	SRR29611990, SRR29611985
Genome browser session<br>(e.g. UCSC)	No longer applicable

Methodology

Replicates	No replicates.
Sequencing depth	ChIP-seq(CENP-A): 12.03Gb raw data, ~4.2X<br>ChIP-seq(INPUT): 18.49Gb raw data, ~6.47X
Antibodies	Phospho-CENP-A (Ser7) rabbit polyclonal antibody (Cat# 2187, Cell Signaling Technology, Beverly, MA, USA)
Peak calling parameters	bowtie2 -p 20 --very-sensitive --no-mixed --no-discordant -k 10 -x rsy.finally.fasta -1 IP1118-2P-CENP-A_1.clean.fq.gz -2 IP1118-2P-CENP-A_2.clean.fq.gz | samtools sort -O bam -@ 10 -o - > rsy.CENP.bam<br>bowtie2 -p 20 --very-sensitive --no-mixed --no-discordant -k 10 -x rsy.finally.fasta -1 INPUT1118-2_1.clean.fq.gz -2 INPUT1118-2_2.clean.fq.gz | samtools sort -O bam -@ 10 -o - > rsy.INPUT.bam<br>macs3 callpeak -t rsy.IP.bam -c rsy.INPUT.bam -g 2.85e9 -f BAM -n rsy -B -q 0.01
Data quality	The centromeres were successfully identified.
Software	Bowtie2 (v2.4.2), MACS3 (v3.0.0b2)
