# Supplementary material for: Telomere-to-telomere genome assembly of a male goat reveals variants associated with cashmere traits
Source: Nat Commun. 2024 Nov 20;15:10041. doi: 10.1038/s41467-024-54188-z (PMC11579321; doi:10.1038/s41467-024-54188-z)
Supplement: Supplementary file 3 — Description of Additional Supplementary Files [file 41467_2024_54188_MOESM3_ESM.pdf]

Description of Additional Supplementary Files

Supplementary Data 1

Description: Summary of sequencing data in this study.

Supplementary Data 2

Description: Statistics of the GV1–GV5 genome assemblies.

Supplementary Data 3

Description: QVs across chromosomes estimated based on NGS kmers.

Supplementary Data 4

Description: Gaps in parental genomes T2T-goat1.0P and T2Tgoat1.0M.

Supplementary Data 5

Description: All the downloaded genome sequencing datasets for global domestic and wild goats.

Supplementary Data 6

Description: Alignments of PacBio long reads of five representative goats in a comparison between T2T-goat1.0 and ARS1 as references.

Supplementary Data 7

Description: Lowcoverage SD regions not covered completely by >1 ONT read.

Supplementary Data 8

Description: Top 15 gene families with expanded gene copies in T2Tgoat1.0 compared to the other 3 assemblies (ARS1, sheep ARS-UI_Ramb_v2.0, and Xinong Saanen Dairy goat).

Supplementary Data 9

Description: Genes annotated on the Y chromosome.

Supplementary Data 10

Description: Primers and TSPY and HSFY copy number using ddPCR.

Supplementary Data 11

Description: T2T-goat1.0 and ARS1 as references are compared for performances of calling SVs based on long reads of five goats.

Supplementary Data 12

Description: The 57 structural variations only occurred in T2T-goat1.0 compared to the PacBio long reads of five goats (Liaoning cashmere, Zhongwei, Jining gray, Boer, and Tibetan goats).

Supplementary Data 13

Description: SVs uniquely identified in the five female goats.

Supplementary Data 14

Description: Top 1% F_{ST} values of selective genomic regions between wild and domestic goats based on SNPs.

Supplementary Data 15

Description: Top 1% F_{ST} values of putative SVs under selection between wild and domestic goats.

Supplementary Data 16

Description: Top 1% XP-CLR values of selective genomic regions between cashmere and noncashmere goats based on SNPs.

Supplementary Data 17

Description: Top 1% F_{ST} values of putative SVs under selection between cashmere

and noncashmere goats.

Supplementary Data 18

Description: Top 12 F_{ST} values of putative SVs under selection between cashmere and noncashmere goats were identified in long reads.

Supplementary Data 19

Description: RNA-seq datasets downloaded from the public database for gene annotation.

Supplementary Data 20

Description: Genome assemblies used for homolog-based gene annotation in T2T-goat1.0.
